# Stress response, behavior, and development are shaped by transposable element-induced mutations in Drosophila

**DOI:** 10.1371/journal.pgen.1007900

**Published:** 2019-02-12

**Authors:** Gabriel E. Rech, María Bogaerts-Márquez, Maite G. Barrón, Miriam Merenciano, José Luis Villanueva-Cañas, Vivien Horváth, Anna-Sophie Fiston-Lavier, Isabelle Luyten, Sandeep Venkataram, Hadi Quesneville, Dmitri A. Petrov, Josefa González

**Affiliations:** 1 Institute of Evolutionary Biology (IBE), CSIC-Universitat Pompeu Fabra, Barcelona, Spain; 2 Institut des Sciences de l'Evolution de Montpellier (UMR 5554, CNRS-UM-IRD-EPHE), Université de Montpellier, Place Eugène Bataillon, Montpellier, France; 3 URGI, INRA, Université Paris-Saclay, Versailles, France; 4 Department of Biology, Stanford University, Stanford, CA, United States of America; Reed College, UNITED STATES

## Abstract

Most of the current knowledge on the genetic basis of adaptive evolution is based on the analysis of single nucleotide polymorphisms (SNPs). Despite increasing evidence for their causal role, the contribution of structural variants to adaptive evolution remains largely unexplored. In this work, we analyzed the population frequencies of 1,615 Transposable Element (TE) insertions annotated in the reference genome of *Drosophila melanogaster*, in 91 samples from 60 worldwide natural populations. We identified a set of 300 polymorphic TEs that are present at high population frequencies, and located in genomic regions with high recombination rate, where the efficiency of natural selection is high. The age and the length of these 300 TEs are consistent with relatively young and long insertions reaching high frequencies due to the action of positive selection. Besides, we identified a set of 21 fixed TEs also likely to be adaptive. Indeed, we, and others, found evidence of selection for 84 of these reference TE insertions. The analysis of the genes located nearby these 84 candidate adaptive insertions suggested that the functional response to selection is related with the GO categories of response to stimulus, behavior, and development. We further showed that a subset of the candidate adaptive TEs affects expression of nearby genes, and five of them have already been linked to an ecologically relevant phenotypic effect. Our results provide a more complete understanding of the genetic variation and the fitness-related traits relevant for adaptive evolution. Similar studies should help uncover the importance of TE-induced adaptive mutations in other species as well.

## Author summary

Transposable elements are fragments of genomic DNA that have the ability to move around the genome by creating new copies of themselves. Although there is evidence suggesting that transposable elements play important roles in genome function and genome evolution, our current understanding is based on the analysis of a subset of all transposable element insertions present in the genome, in only a few natural populations. Advances in genome sequencing and bioinformatics tools allow us now to investigate all transposable element insertions in genomes across continents. In this work, we analyzed the age, size, and frequency of 1,615 transposable elements in 91 samples from 60 worldwide natural populations of the model species *Drosophila melanogaster*. The genomic regions surrounding 84 transposable elements indicate that they could have an adaptive effect for the flies that carry them. These insertions are located nearby genes involved in stress response, behavior and development suggesting that these biological processes are affected by transposable element insertions.

## Introduction

Understanding how organisms adapt to local environmental conditions requires identifying the loci and the phenotypic traits potentially targeted by natural selection, which should also provide critical knowledge for how organisms will respond to environmental change [1–3]. Organisms from plants to humans harbor genetic variation within and among populations that allows them to adapt to diverse local environments [4–6]. Genome scans for selection have almost exclusively focused on identifying single nucleotide polymorphisms (SNPs). However, while the role of other types of genetic variants, such as transposable element (TE) insertions and segmental duplications, in local adaptation has been suggested, these variants are often poorly characterized [7–10]. This is mainly due to technical limitations: short-read sequencing technologies make TE discovery and accurate genotyping difficult. However, deciphering the genetic basis of adaptation requires comprehensive knowledge of these other types of genetic variants, as there is evidence that they are important contributors to adaptive variation [9, 11, 12].

TEs are mobile DNA fragments that constitute a substantial albeit variable proportion of virtually all the genomes analyzed to date [13, 14]. TEs can create a variety of mutations from gene disruption to changes in gene expression and chromosome rearrangements [14, 15]. Although the majority of TE-induced mutations are deleterious or neutral, there are multiple instances in which individual TE insertions have been shown to play a role in adaptive evolution [10–12, 16]. In humans, *MER41* insertions, a family of endogenous retroviruses, have dispersed interferon-inducible enhancers that promote the transcription of innate immunity factors [17]. In *Drosophila melanogaster*, the insertion of an *Accord* retrotransposon in the upstream region of *Cyp6g1* gene leads to transcript up-regulation and increased resistance to several insecticides [18, 19].

However, only a few genome-wide screens have tried to systematically assess the role of TEs in adaptive evolution. In humans, the only screen so far focused on the analysis of a particular TE family, LINE-1 elements, and found that a fraction of these elements showed signatures of positive selection [20]. In *D*. *melanogaster*, genome-wide screens were initially performed based on a PCR-approach that only allowed studying a subset of all the euchromatic TEs present in the reference genome [7, 8, 21]. Later on, a genome-wide screening using bioinformatics approaches was also performed in this species [22]. In *Arabidopsis thaliana*, genome-wide analysis of TE insertions revealed that TEs affect nearby gene expression and local patterns of DNA methylation, with some of these insertions likely to be involved in adaptation [23, 24]. Thus, while at the moment limited to species with good TE sequence annotations and genome datasets, genome-wide screens for putatively adaptive insertions are a promising strategy to identify genetic variants underlying adaptive evolution [25].

*D*. *melanogaster* is to date one of the best model systems to identify the genetic and functional basis of adaptive evolution. Originally from sub-tropical Africa, *D*. *melanogaster* has adapted in recent evolutionary time to a wide-range of environmental conditions [26, 27]. Indeed, there are hundreds of genome sequences available from worldwide populations [28, 29]. This species has one of the best functionally annotated genomes, which facilitates the identification of traits under selection [30]. In addition, TE annotations in the *D*. *melanogaster* reference genome continue to be updated by the research community [31–33].

**Fig 1:**
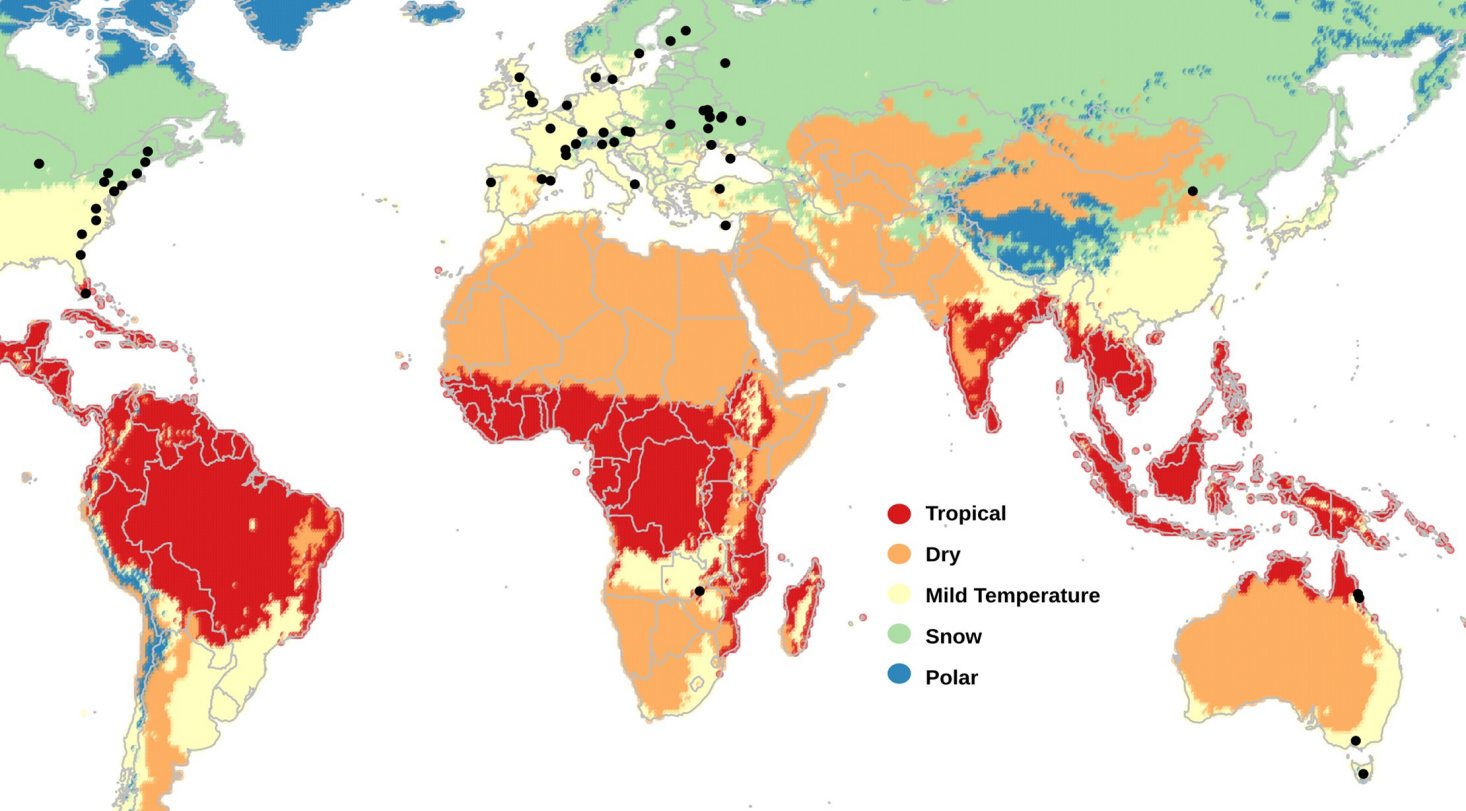
Worldwide distribution of *D*. *melanogaster* populations used in this study. Location of the 39 European, 14 North American, five Australian, one Asian, and one African population analyzed in this work. Note that the location of some populations overlap in the map. For more details, see S1 Table. Colors indicate the five major Köppen climate zones [114].

In this work, we screened 303 individual genomes, and 83 pooled samples (containing from 30 to 702 genomes each) from 60 worldwide natural *D*. *melanogaster* populations to identify the TE insertions most likely involved in adaptive evolution (Fig 1). We focused on TE insertions annotated in the reference genome because for these insertions we can analyze their age and length, which are informative about their population dynamics [21, 34, 35]. In addition to the age and the size of the 1,615 TEs analyzed, we calculated five different statistics to detect potentially adaptive TE insertions. The GO enrichment analysis of the genes located nearby our set of candidate adaptive insertions pinpoint response to stimulus, behavior, and development as the traits more likely to be shaped by TE-induced mutations. Consistent with these results, genes located nearby our set of candidate adaptive TEs are significantly enriched for previously identified loci underlying stress- and behavior-related traits. Overall, our results suggest a widespread contribution of TEs to adaptive evolution in *D*. *melanogaster* and pinpoint relevant traits for adaptation.

## Results

### Natural populations of *D*. *melanogaster* contain hundreds of polymorphic TEs at high population frequencies

To identify TEs likely to be involved in adaptation, we looked for TEs present at high population frequencies, and located in genomic regions with high recombination rates (see Material and Methods). We expect TEs that increase the fitness of their carriers to be present at high frequency in the population(s) where adaptation took place [36–39]. In addition, among all the TEs present at high frequencies, TEs located in regions with high recombination rates are less likely to have increased in frequency neutrally compared with TEs located in low recombination regions. This is so because the efficiency of selection in genomic regions with low recombination rates tends to be lower due to the increase in noise generated by linked selection such as background selection and recurrent selective sweeps [35, 40]. Moreover, TEs located in low recombination regions are more likely to be linked to an adaptive mutation rather than being the causal mutation [36–38].

**Fig 2:**
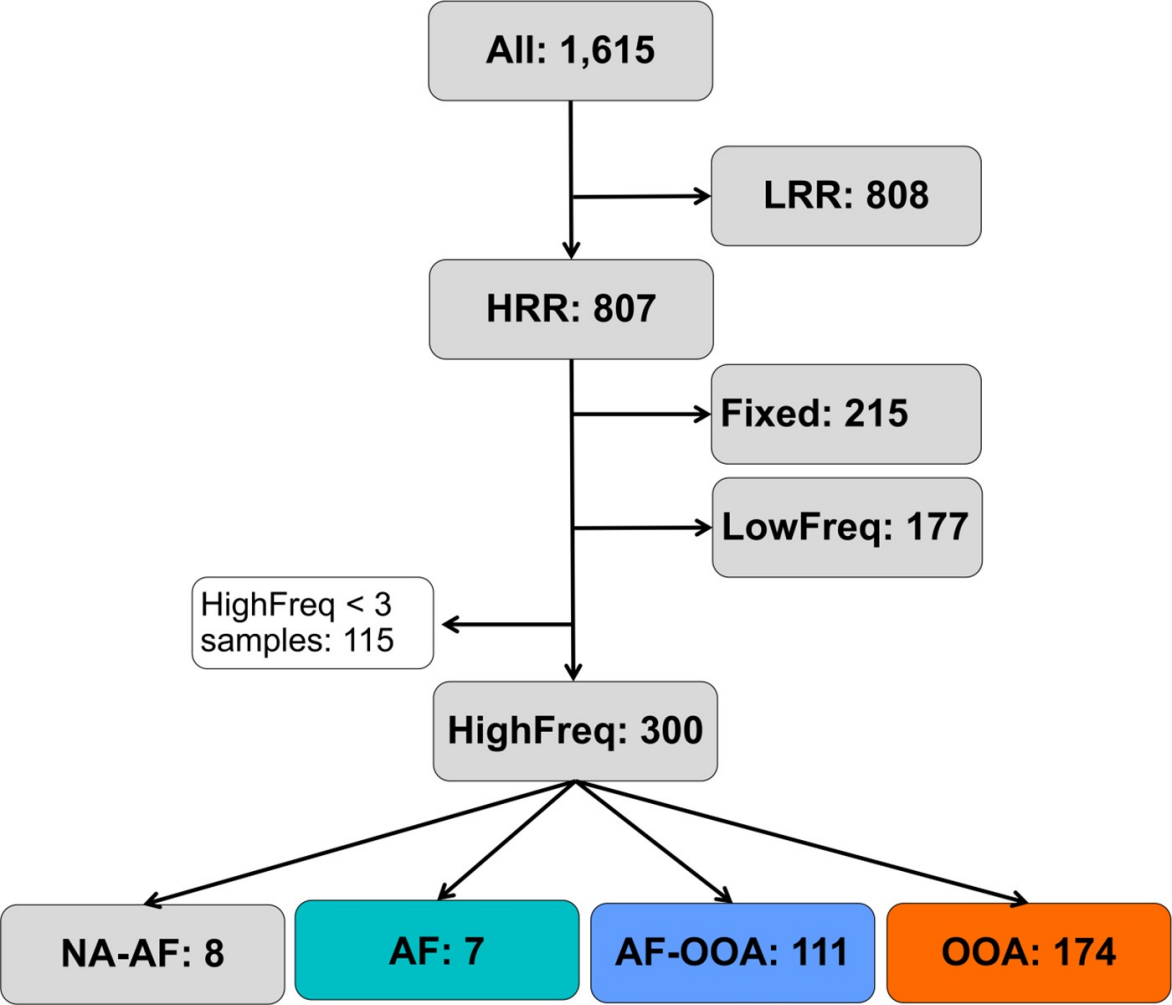
Workflow showing the main steps applied for identifying TEs present at high frequencies in high recombination regions in the *D*. *melanogaster* genome. LRR: TEs located at low recombination rate regions. HRR: TEs located at high recombination rate regions. Fixed: HRR TEs at frequencies > 95% in all populations. LowFreq: low frequency HRR TEs (frequencies < 10% in all samples). HighFreq: high frequency HRR TEs (frequencies < 95% in all samples and at >10% frequency in at least three samples). HighFreq TEs were further classified according to their frequency in African (AF) and/or out-of-Africa (OOA) populations: AF: TEs at high frequency only in the African population; AF-OOA: TEs at high frequency in Africa and out-of-Africa populations; OOA: TEs at high frequency in out-of-Africa populations and low frequency in the African population and NA-AF: TEs present at high frequency in out-of-Africa populations but for which we have no data for the African population.

We first estimated population frequencies, using *T-lex2* [33], for 1,615 reference TE insertions in 91 samples from 60 worldwide natural populations: 39 European, 14 North American, five Australian, one Asian, and one African population collected in the ancestral range of the species (Fig 1 and S1 Table) (see Material and Methods). *T-lex2* estimates both the presence and the absence of insertions [33]. Thus, for individual genomes, *T-lex2* can distinguish between homozygous and heterozygous insertions. To estimate TE frequencies from pooled samples, *T-lex2* takes into account the number of reads supporting the presence and the number of reads supporting the absence of that particular insertion. We classified the 1,615 TEs based on their population frequencies obtained with *Tlex2* [33], and on their genomic location in high or low recombination regions (Fig 2, S2 Table, see Material and Methods). 808 of the 1,615 TEs were present in regions with low recombination rate. Most of these TEs (79%, 640 out of 808 TEs) were fixed, defined here as being present at > 95% frequency in all samples, in all the populations analyzed. Among the 807 TEs located in regions with high recombination rates, 215 were fixed and 177 were present at low frequencies (LowFreq), defined here as being present at ≤ 10% frequency in each of the analyzed samples (Fig 2). Note that the percentage of fixed TEs in high recombination regions is significantly lower than the percentage in low recombination regions (27% vs 79% respectively, Chi-squared p-value = 2.2e-16), as expected if the efficiency of selection is lower in low recombination regions, and slightly deleterious TEs reached fixation neutrally [35, 40]. Finally, 300 of the 807 TEs located in high recombination regions were present at high frequencies (HighFreq), defined here as being present at < 95% frequency overall and at >10% frequency in at least three samples (Fig 2, S1 Fig).

We further classified these 300 TEs according to their frequency in African (AF) and/or out-of-Africa (OOA) populations: seven TEs were only present at high frequencies in the African population analyzed (AF), 111 were present at high frequencies both in African and in the out-of-Africa populations (AF-OOA), and 174 were present at high frequencies only in the out-of-Africa populations (OOA, Fig 2). TEs present at high frequencies both in African and out-of-Africa populations are more likely to be involved in global (shared) adaptations, while TEs present only in African or only in out-of-Africa populations could be involved in local adaptation. Overall, we identified 300 polymorphic TEs present at high frequencies and located in high recombination regions of the genome, which could have increased in frequency due to positive selection. However, it is also possible that some or many of these 300 TEs have increased in frequency neutrally.

### Age and length of TEs present at high frequencies in regions with high recombination are consistent with a putatively adaptive role of these insertions

In addition to the population frequency, the age of a TE insertion can be informative about whether a TE is more likely to be adaptive, neutral, or deleterious. A young TE present at high population frequencies is more likely to have increased in frequency due to recent positive selection, while old TEs present at high population frequencies might have slowly drifted to high frequency [21, 25]. Note that it is entirely possible that such old TEs did increase in frequency due to positive selection and have been maintained by balancing selection since then [41]. Nonetheless, in this paper we primarily focus on the identification of the subset of TEs that are most likely to be adaptive and are willing to tolerate potentially high false negative rates.

We estimated the age of all the TEs annotated in the reference genome using a phylogenetic approach (5,416 TEs, see Material and Methods). Briefly, we estimated the unique number of substitutions shared between the two closest TEs assuming that they all derived from a common ancestral TE within each family. We compared our TE age estimates with previously available data for 437 TEs [21, 42]. Among the 417 TEs present in the two datasets, there are 10 TE insertions in our dataset that according to the TE age distributions were outliers (showed much higher age values estimates, S2A Fig). When we removed these 10 data points the correlation between the age estimates from the two studies was high (*r*^*2*^: 0.71, p-value < 2.20e-16, S2B Fig). Note that the TE age estimates obtained by these methods depend on the dataset used for generating the phylogenies, which differ between the two studies (437 TEs vs 5,416 TEs, S2 Fig).

**Fig 3:**
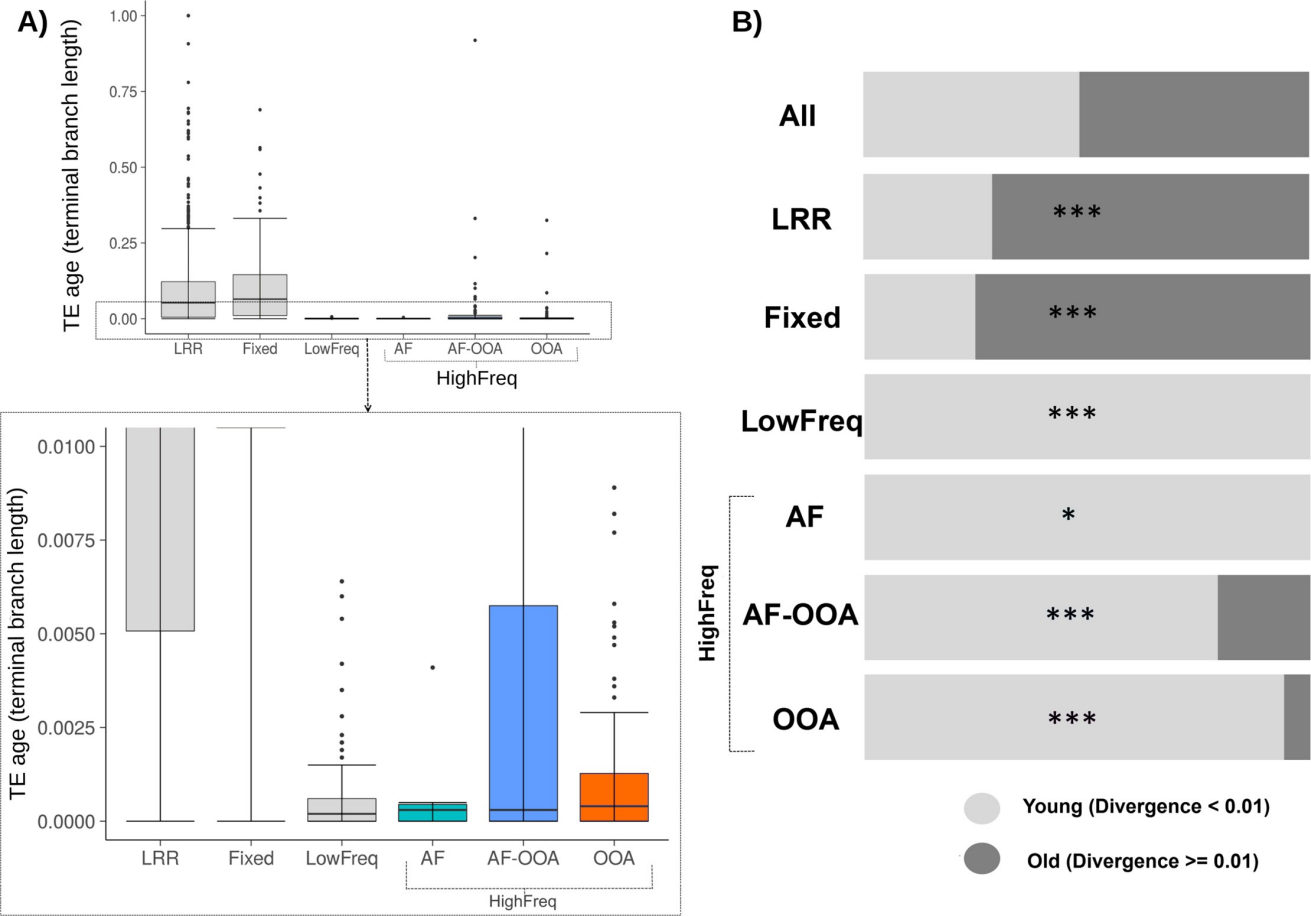
TE age of the different frequency groups. **A)** Top: Boxplots showing the distribution of TE age (terminal branch length) values for each of the categories. Bottom: Zoomed-in version of the boxed area showing the lowest values of the TE age distribution. **B)** Proportion of young (age < 0.01) and old (age ≥ 0.01) TEs in each category. * p-value < 0.05, *** p-value < 0.001 from Chi-square test.

We compared the TE age distributions between the different frequency groups, and we further classified TEs as “young” or “old” insertions according to whether the estimated terminal branch length was < 0.01 or ≥ 0.01, respectively (see Material and Methods). As mentioned above, most of the TEs in low recombination regions are fixed. Accordingly, we found that TEs present in low recombination regions and Fixed TEs in high recombination regions showed similar age distributions (Wilcoxon test, p-value = 0.321, Fig 3A) and contained a large proportion of old TEs, 71% and 75% respectively, as expected if these two datasets contain mostly neutral TEs (Fig 3B, Table 1). The age distribution of these two groups was different from the LowFreq and the HighFreq groups overall (Wilcoxon test, p-value < 2.20e-16, Fig 3A).

**Table 1:** Age distribution of TEs belonging to the different population frequency categories.

TE category			Old	Young	P-value*	Enrichment
All			608 (48%)	661 (52%)	-	-
LRR			480 (71%)	198 (29%)	2.20e-16	Old TEs
HRR	All		128 (22%)	463 (78%)	-	-
	Fixed		100 (76%)	31 (24%)	2.33e-14	Old TEs
	LowFreq		0 (0%)	177 (100%)	2.20e-16	Young TEs
	HighFreq	All	28 (10%)	255 (90%)	2.20e-16	Young TEs
		NA-AF	0 (0%)	8 (100%)	6.22e-03	Young TEs
		AF	0 (0%)	7 (100%)	6.22e-03	Young TEs
		AF-OOA	19 (19%)	82 (81%)	4.63e-12	Young TEs
		OOA	9 (5%)	158 (95%)	2.20e-16	Young TEs

*P-values are from Chi-square tests comparing TEs at each category with the expectations based on “All TEs”. Note that TEs without age or category classification were excluded from this analysis.

We found that all LowFreq TEs were young TEs (Fig 3B, Table 1). This result is consistent with LowFreq TEs being slightly deleterious mutations that have not been yet removed from populations by purifying selection. Finally, the three subgroups of HighFreq TEs contained mostly young TEs (Fig 3B, Table 1).

**Fig 4:**
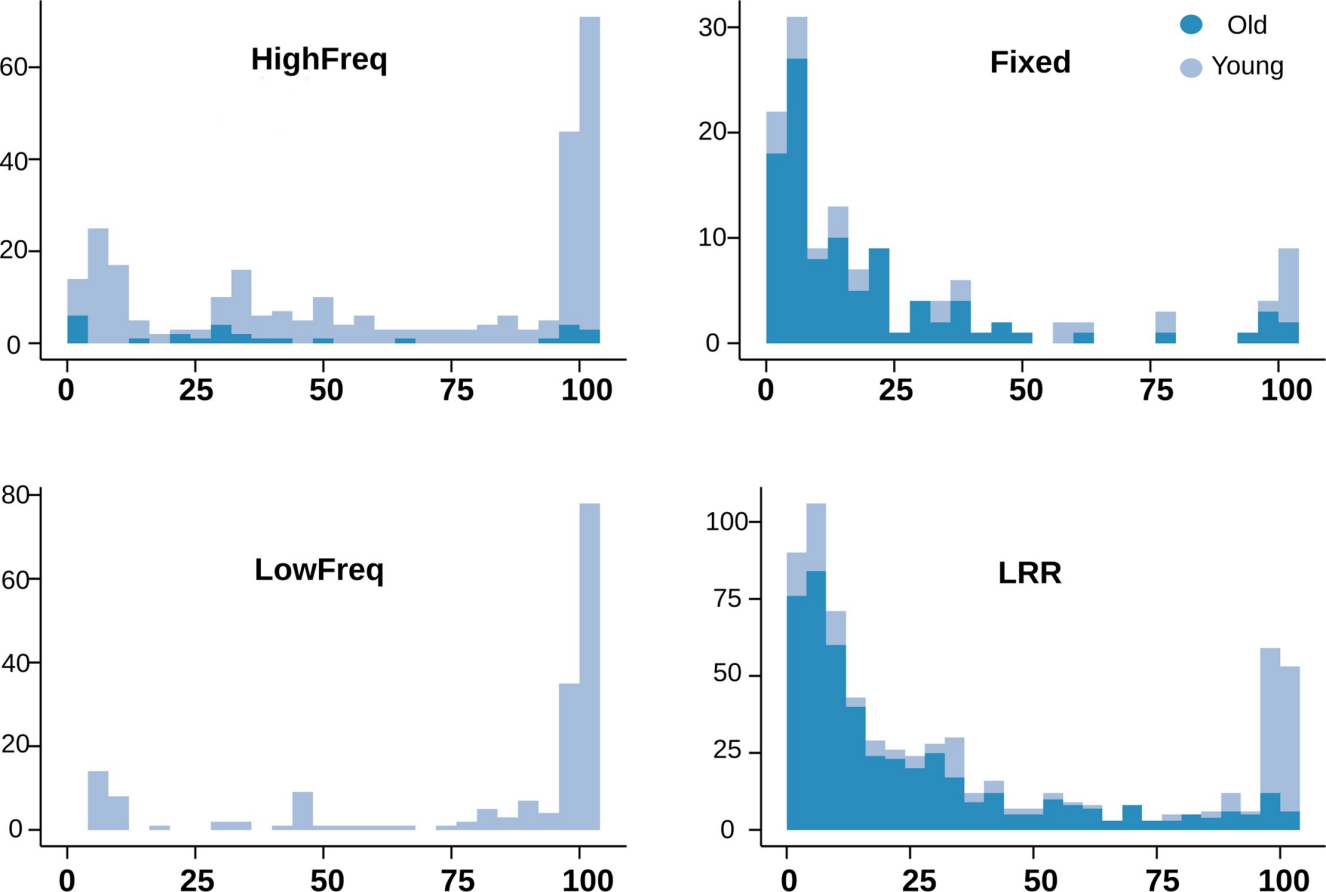
Number of TEs at different TE length ratios (%). Bars indicate number of TEs (vertical axis) per bin of TE Length Ratio (%) (horizontal axis) and color shade indicates the proportion of young and old TEs in each bin.

The length of a TE can also be informative about whether a TE is more likely to be adaptive, neutral, or deleterious. Because longer TEs are more likely to act as substrates for ectopic recombination leading to deleterious rearrangements, if a TE is long but it is present at high population frequencies, it is more likely to be adaptive [16, 34, 43]. In contrast, shorter TEs are both more likely to be nearly neutral in their selective effect due to lower rate of ectopic recombination among shorter homologous sequences, and in addition more likely to be older and thus shorter because of the high rate of DNA loss in Drosophila [44]. We used the TE length ratio, calculated as the proportion of the length of the TE insertion regarding the length of the canonical family sequence, as a proxy for measuring the relative length of the TEs in each group. We found statistically significant differences between the HighFreq and the other three TE groups: LowFreq, Fixed, and TEs in low recombination regions (S3A Table). In particular, HighFreq and LowFreq TEs showed distributions of TE Length Ratio shifted upwards (median: 59.3 and 80.4, respectively), while the distributions of Fixed TEs and TEs in low recombination regions are shifted downwards, showing a predominance of shorter TEs (mean: 16.2 and 30.7, respectively) (Fig 4 and S3A Table). No differences in the TE length ratio among the HighFreq TEs subgroups were found (Kruskal Wallis test, p = 0.062) (S3A Table). Similar results were obtained when we controlled for the age of the insertions (S3A Fig and S3B and S3C Table), and the class identity of the insertions (S3B Fig and S3D, S3E and S3F Table) in each frequency category.

When considering both age and length of the TEs across different categories, we found that Fixed TEs and TEs in low recombination regions show a predominance of older and truncated TEs (Fig 4), which is consistent with old TE insertions that have reached fixation through processes other than positive selection. On the other hand, the HighFreq and LowFreq groups contain mostly large and young TEs (Fig 4). In the case of LowFreq TEs, these results are consistent with the hypothesis that low frequency TEs could be recent insertions that purifying selection still did not have time to eliminate. Finally, young and large HighFreq TEs support the hypothesis of the presence in this group of a large number of recent putatively functional insertions that have rapidly increase in frequency due to the action of positive selection.

### TEs present at high frequencies in high recombination regions showed different signatures of positive selection

To test whether HighFreq TEs showed signatures of positive selection, we used two different approaches: we looked for signatures of selective sweeps in the regions flanking the candidate adaptive TEs, and we looked for evidence of population differentiation between populations located at the extremes of latitudinal clines in three continents: Europe (EU), North America (NA), and Australia.

**Fig 5:**
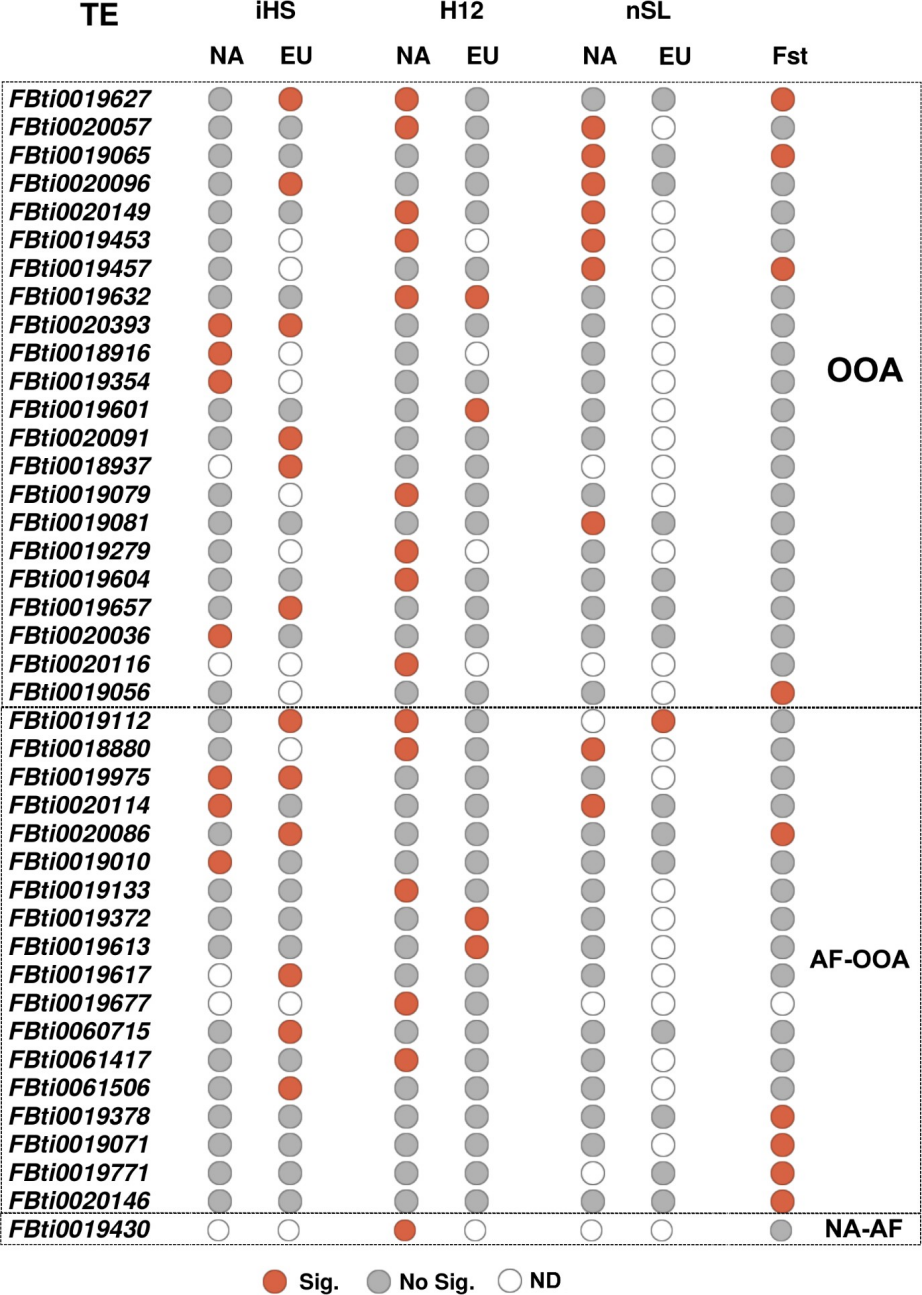
HighFreq TEs with signals of selection. 41 HighFreq TEs showing at least one signal of selection either or both in the selective sweep tests (*iHS*, *H12* or *nSL*, 36 TEs) or the population differentiation test (F_**ST**_, 9 TEs). Red and grey circles indicate statistical significance for each TE at each test and population (Significant and No significant, respectively). Empty circles (ND) indicates that the test could not be calculated.

To look for signatures of selective sweeps in the vicinity of the candidate TE insertions, we used three different haplotype-based methods in order to identify different signals of selection: (i) the *iHS* test mainly detects events of hard sweeps [45], (ii) the *H12* test detects both hard and soft sweeps [46], and (iii) the *nSL* test detects sweeps under different scenarios, and it is more robust to recombination rate variation [47]. We independently applied these tests to two datasets: one dataset containing 141 strains from the Raleigh population in NA, and a second dataset containing 158 strains from four different populations in EU. Note that EU populations do not show latitudinal population structure, and thus we analyzed them together [48] (see Material and Methods). Overall, we were able to calculate at least one test, in at least one of the two continents, for 202 of the 300 HighFreq TE insertions (S4 Table). To determine the significance of *iHS* and *nSL* values, we compared them with the distribution of values obtained from neutral SNPs, while for *H12* we selected the top 15% values (see Material and Methods). Overall, 36 TEs showed evidence of selection (Fig 5 and S5 Table). The three tests identified similar numbers of significant TEs (Chi-square test, p-value = 0.350, S4 Table), however the overlap between the TEs identified by the different tests was low (S4A Fig). These results suggest that these 36 TEs could be evolving under different selective scenarios, including both hard and soft sweeps.

We also tested whether the signals of selection differ among continents. For 31 out of the 36 TEs that showed signatures of selection, we had data from NA and EU populations. Only six of these 31 TEs showed evidence of selection in both continents while the other 25 TEs were significant only in NA or only in EU, suggesting that the signatures of selection could be continent specific (S4B Fig). However, these results could also be due to differences in the ability to detect selection in the two continents due to the differences in the two datasets used. Finally, while *iHS* and *nSL* identified similar numbers of TEs in the two continents, *H12* identified more significant TEs in NA (Chi-square test, p-value = 0.032, S4 Table).

Besides selective sweeps, we also looked for evidence of population differentiation using the pairwise F_ST_ estimator of Weir & Cockerham (1984) [49]. We performed six pairwise comparisons among latitudinal distant populations: two populations in EU, two in NA, and two in Australia (see Materials and Methods). We could estimate F_ST_ for 254 of the 300 HighFreq TE insertions (S6 Table). To determine the significance of F_ST_ values, we compared them with the distribution of values obtained from neutral SNPs in each pair of populations (see Material and Methods). 78 TEs showed significant F_ST_ values, and we further filtered them by keeping only those that were significant in more than one pairwise comparison and consistently present at high frequencies in populations located in high latitudes or in low latitudes (concordant F_ST_) (see Material and Methods). After this filtering step, nine TEs were significant (S5 Fig). Four of these nine TEs were also identified as being under positive selection by at least one haplotype-based test (Fig 5).

Overall, we could calculate at least one statistic for 273 HighFreq TEs, and 41 of them showed evidence of positive selection (Fig 5, S4 Table). TEs present at high frequencies both in African and in the out-of-Africa populations (AF-OOA), and TEs present at high frequencies only in the out-of-Africa populations (OOA) showed similar percentage of TEs with evidence of selection, 18/103 (17.5%) and 22/154 (14.2%) respectively (Chi-square, p-value = 0.488, S4 Table), suggesting that both datasets could be enriched for adaptive TEs. Note that, nine of these 41 TEs were previously found to show evidence of positive selection (Table 2).

**Table 2:** 84 reference TE insertions showed evidence of selection. The 62 TEs identified in this work are listed at the top of each frequency category, followed by TEs identified in other studies. Note that for 11 of the 62 TEs there was previous evidence suggesting that they were evolving under positive selection.

TE category	Flybase ID	Evidence of selection	Reference	GO enrichment/ Gene association
OOA	FBti0018916	iHS	This work	-
	FBti0018937	iHS	This work	RtS/ olfactory
	FBti0019056	F_ST_ / CSTV	This work/ [51]	RtS
	FBti0019065	F_ST_, nSL / fTE / CSTV	This work / [7]/ [51]	RtS/ xenobiotic
	FBti0019079	H12	This work	RtS
	FBti0019081	nSL	This work	RtS
	FBti0019279	H12	This work	RtS/ alcohol, olfactory
	FBti0019354	iHS / Allele age	This work/ [21]	- /alcohol
	FBti0019453	H12, nSL	This work	RtS/circadian
	FBti0019457	F_ST_/nSL	This work	-
	FBti0019601	H12	This work	-/ xenobiotic
	FBti0019604	H12	This work	RtS/ alcohol, heavy metal, olfactory
	FBti0019627	F_ST_, iHS, H12/ iHS / Phenotypic	This work/ [7]/ [52]	RtS/ xenobiotic, diapause
	FBti0019632	H12	This work	RtS
	FBti0019657	iHS	This work	RtS
	FBti0020036	iHS	This work	RtS/ agressiveness, hypoxia, olfactory
	FBti0020057	H12 / nSL	This work	- / immunity, xenobiotic, diapause
	FBti0020091	iHS	This work	-
	FBti0020096	iHS/ nSL	This work	-
	FBti0020116	H12	This work	RtS/ olfactory
	FBti0020149	H12, nSL / Allele age	This work/ [21]	- / olfactory
	FBti0020393	iHS	This work	RtS/heavy metal
	FBti0019360	F_ST_	[53]	-
	FBti0020125	Allele age	[21]	RtS/olfactory
	FBti0019386	CL test, TajimaD, Phenotypic	[54]	RtS
	FBti0019985	TajimaD, iHS, H12, Phenotypic	[55]	RtS/ diapause
	FBti0020155	Phenotypic	[56]	RtS/ immunity, starvation, alcohol
	FBti0020046	Allele age	[21]	-/ immunity
AF-OOA	FBti0018880	H12, nSL / iHS / Phenotypic	This work / [7] [57]/ [58]	- /immunity, xenobiotics, alcohol, circadian, starvation, heat-shock
	FBti0019010	iHS / F_ST_	This work/ [53]	RtS
	FBti0019071	F_ST_	This work	-
	FBti0019112	iHS, H12, nSL	This work	RtS/ alcohol, olfactory, starvation
	FBti0019133	H12	This work	RtS/ agressiveness
	FBti0019372	H12	This work	RtS/ olfactory, pigmentation
	FBti0019378	F_ST_	This work	RtS
	FBti0019613	H12	This work	RtS
	FBti0019617	iHS	This work	RtS/ alcohol, diapause
	FBti0019677	H12	This work	-/starvation, agressiveness
	FBti0019771	F_ST_	This work	-
	FBti0019975	iHS	This work	-
	FBti0020086	F_ST_, iHS / Allele age	This work/ [21]	RtS/ circadian, xenobiotic
	FBti0020114	iHS, nSL	This work	-
	FBti0020146	F_ST_	This work	RtS
	FBti0060715	iHS	This work	RtS
	FBti0061417	H12	This work	RtS/ heavy metal
	FBti0061506	iHS	This work	RtS/ hypoxia, immunity, olfactory, xenobiotics
	FBti0019276	CSTV	[51]	RtS
	FBti0019344	F_ST_	[53]	RtS
	FBti0019564	TajimaD	[22]	RtS
	FBti0019611	CSTV	[51]	Nsd, locomotion, chemotaxis / olfactory, pigmentation, alcohol, diapause
	FBti0019082	TajimaD	[22]	RtS/ starvation
	FBti0060443	CSTV	[51]	RtS/ alcohol
	FBti0019170	fTE / Phenotypic	[7]/ [59]	RtS/ olfactory
NA-AF	FBti0019430	H12 / TajimaD / iHS, fTE / Alllele age / Phenotypic	This work/ [22]/ [7]/ [21]/ [60] [61]	-/ immunity, hypoxia
	FBti0019200	Allele age	[21]	RtS/ starvation
LowFreq	FBti0020082	Allele age	[21]	RtS
	FBti0061742	TajimaD	[22]	-
Fixed	FBti0059674	Young&Long	This work	-/ alcohol, cold, heavy-metal, olfactory, pigmentation, xenobiotics
	FBti0019153	Young&Long	This work	-
	FBti0019149	Young&Long	This work	-
	FBti0059794	Young&Long	This work	-/ heavy-metal, olfactory
	FBti0019355	Young&Long	This work	-/ xenobiotic
	FBti0019590	Young&Long	This work	RtS, Development / pigmentation
	FBti0019191	Young&Long	This work	-/ alcohol, olfactory
	FBti0020098	Young&Long	This work	-/ alcohol
	FBti0020101	Young&Long	This work	-/ alcohol
	FBti0020015	Young&Long	This work	-/ pigmentation, diapause, hypoxia, oxidative, starvation, xenobiotic, alcohol, oxidative, xenobiotics
	FBti0020013	Young&Long	This work	-/ alcohol, olfactory, heavy-metal, pigmentation
	FBti0019199	Young&Long / Allele age	This work/ [21]	RtS/ alcohol, pigmentation
	FBti0018940	TajimaD	This work	-
	FBti0020147	TajimaD	This work	-
	FBti0060295	TajimaD	This work	-
	FBti0061024	TajimaD	This work	-
	FBti0062854	TajimaD	This work	-
	FBti0062980	TajimaD	This work	-
	FBti0063022	TajimaD	This work	-
	FBti0063801	TajimaD	This work	-/ alcohol, diseccation, pigmentation
	FBti0060388	TajimaD	This work/ [22]	RtS
	FBti0060479	TajimaD	[22]	RtS
	FBti0062283	TajimaD	[22]	RtS/ immunity, alcohol
	FBti0063191	TajimaD	[22]	RtS/ alcohol, diapause, immunity, oxidative, starvation, xenobiotic
	FBti0019655	TajimaD	[22]	-
	FBti0020329	TajimaD	[22]	RtS/ hypoxia
	FBti0059793	TajimaD	[22]	- /immunity, oxidative, starvation, alcohol, hypoxia

CSTV: Correlation with spatio-temporal variables. RtS: response to stimulus, Nsd: Nervous system development.

### A subset of the Fixed TEs in high recombination regions are also candidate adaptive insertions

Besides polymorphic insertions, we also analyzed whether any of the Fixed TE insertions could be also considered as candidate adaptive insertions. To do this, we first identified Fixed TEs that were (i) young, and thus likely to have reached fixation rapidly, and (ii) long insertions (>50% of the canonical length), and thus not likely to have reached fixation neutrally. 12 of the 215 Fixed insertions were both young and long TEs and we considered them as candidate adaptive insertions (Table 2). Second, we estimated Tajima´s D genome-wide [50] and also considered as candidate adaptive Fixed TEs those that are nearby windows with significantly negative Tajima´s D values, suggesting that they have increased in frequency due to positive selection [22] (S7 Table) (see Material and Methods). Thus, overall we identified 21 out of the 215 Fixed TEs in high recombination regions as candidate adaptive insertions (Table 2).

### Candidate adaptive TEs are associated with genes involved in stress response, behavior, and development

**Fig 6:**
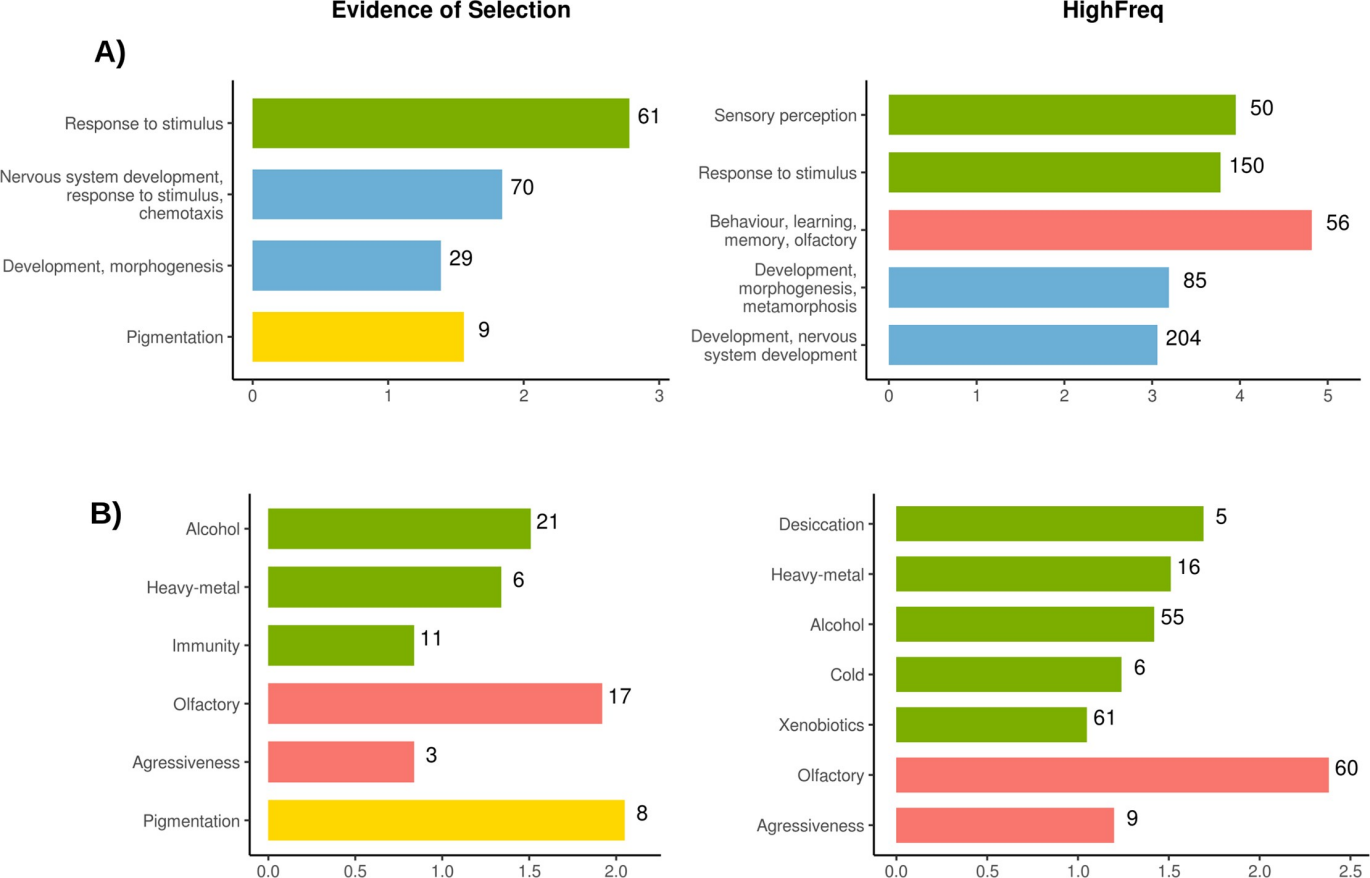
Functional enrichment analysis of genes nearby TEs showing evidence of selection (in this or previous works) and HighFreq TEs. Bar colors indicates similar biological functions of the DAVID clusters (**A**) and the fitness-related traits (**B**): Green: stress response, Red: behavior, Blue: development Yellow: pigmentation. **A)** Significant gene ontology clusters according to DAVID functional annotation tool (enrichment score > 1.3). For genes nearby HighFreq TEs, only top five clusters are showed. The horizontal axis represent DAVID enrichment score (see S8A and S8B Table for details). **B)** Significantly overrepresented fitness-related genes according to previous genome association studies. All FDR corrected p-values < 0.05, Chi-square (χ^2^) test (see S10A and S10B Table for details). The horizontal axis represents the log_10_(χ^2^). In both, A) and B), numbers nearby each bar indicate total number of genes in that cluster/category.

We used the GO terms of genes nearby candidate adaptive TEs to test whether they were enriched for any biological processes. Besides, the 62 TEs identified in this work, we also consider 22 TEs that have been previously identified as candidate adaptive TEs based on different approaches such as Tajima’s D, and the age of allele neutrality test (Table 2). In total, we analyzed 111 genes nearby 84 TEs (Table 2, S8A Table). We found four significant clusters (enrichment score > 1.3) according to DAVID [62, 63] functional annotation tool related with nervous system development, response to stimulus, and pigmentation (Fig 6A, S8A Table). We then analyzed whether the 363 genes nearby the 300 HighFreq TEs were enriched for similar biological processes (see Material and Methods). We identified 20 significant clusters (S8B Table). Among clusters showing the highest enrichment scores we also found GO terms related with response to stimulus and development, and with behavior and learning (Fig 6A). Finally, genes nearby OOA and AF-OOA TEs were also enriched for similar biological functions (S6 Fig, S8C and S8D Table). Note that the behavior-related clusters slightly differed among the datasets: genes nearby HighFreq TEs and AF-OOA TEs were enriched for olfactory genes, and genes nearby OOA TEs for circadian and locomotor behavior genes (Fig 6 and S6 Fig). Note that we discarded that these GO enrichments were due to insertion biases of families enriched in our dataset (S8E and S8F Table, see Material and Methods).

To gain more insight into the function of genes nearby the candidate adaptive TEs, we looked whether they were previously described as candidate genes for several fitness-related traits (S9 Table, see Material and Methods). Among the 111 genes nearby the 84 candidate adaptive TEs, 41 have previously been identified as candidates for stress-related phenotypes, including 21 genes associated with alcohol exposure, six with heavy-metal, and 11 with immunity (Fig 6B, S10A Table). In addition, we also found enrichment of genes related with behavioral phenotypes such as olfaction and aggressiveness, and with pigmentation (Fig 6B, S10A Table). Similar enrichments were found for genes located nearby the 300 High Freq TEs (Fig 6B, S10B Table) and for the genes located nearby the OOA and the AF-OOA datasets (S6 Fig, S10C and S10D Table). Note that among the 363 genes nearby HighFreq TEs, 171 genes (hypergeometic test p-value = 1.00e-05) have previously been identified as candidates for stress-, behavior- and other fitness-related traits (Fig 6B, S9 Table). Indeed, 148 of the 363 genes (hypergeometic test p-value = 6.00e-03) have previously been identified as candidates, if we only consider stress-related phenotypes (Fig 6B, S9 Table).

Overall, we found that genes nearby the 300 HighFreq TEs are enriched for similar biological processes as genes nearby a dataset of 84 TEs with evidence of positive selection: response to stimulus and development (Fig 6A, S8 Table). Moreover, 47% of the genes nearby the 300 HighFreq TE dataset have previously been identified as candidate genes for several stress-, behavior- and/or fitness-related traits (Fig 6B, S10 Table).

### Candidate adaptive TEs correlate with the expression of nearby genes

We tested whether there was a correlation between the presence of the candidate adaptive TEs and the expression of nearby genes using the *Matrix eQTL* package [64]. We used gene expression data from Huang *et al*. [65] and *T-lex2* annotations for 140 DGRP lines in order to determine whether the presence of a TE was correlated with the expression level of the nearby genes (< 1kb). We calculated correlations for 638 TEs located at high recombination regions and we found that 19 of them showed significant eQTL associations (S11 Table). TEs present at high frequencies contained more significant eQTLs than expected (38% vs 11%, Chi-Square test, p-value < 0.0001) (Table 3). We observed the same significant tendency when considering only positive correlations (the presence of the TE correlates with increased expression of the nearby gene) or only negative correlations (the presence of the TE correlates with reduced expression of the nearby gene) (Table 3). These results remained significant after FDR correction (50% vs 11% expected, Chi-Square test, p-value < 0.0001, Table 3). Of the 19 TEs showing significant eQTL associations, 11 also showed signatures of selection (S11 Table).

**Table 3:** Correlation between TE presence and expression level of nearby genes.

TEs		HighFreq		Fixed		LowFreq		Private	
**All TEs analyzed**		70	11%	192	30%	376	59%	25	4%
**Significant TEs**	**All**	19	38% (***)	12	24%	19	38%	4	8%
	**Positive correlation**	15	37% (***)	11	27%	15	37%	4	10% (*)
	**Negative correlation**	11	32% (***)	8	24%	15	44%	3	9% (*)
	**FDR<0.05**	5	50% (***)	0	0%	5	50%	0	0%

Number of TEs located in high recombination regions for which correlations were calculated (All TEs analyzed), and number of TEs with significant correlations for each frequency group are given (Significant TEs). Frequency groups were determined based on their frequency in the DGRP population. LowFreq TEs were further classified as Private if only one strain contained the TE. Note that TEs are classified as fixed if they are present in > 95% of the strains analyzed, thus for some of these TEs there could be strains that do not contain the insertion. Percentages regarding the total number of TEs in that frequency category are also given. Chi-square test * p-value < 0.05 and *** p-value < 0.0001.

We finally checked whether private TEs (those present in only one DGRP strain according to *T-lex2*) were also present among the significant eQTL as expected by the “rare alleles of large effect” hypothesis [66]. We found a small, but still significant set of private TEs with significant correlation with the expression of nearby genes (10% and 9% vs 4% expected, Chi-Square test, p-value < 0.050) (Table 3), which is in agreement with previous reports [67].

### Genomic location, order, and family enrichment of TEs present at high frequencies in high recombination regions

**Fig 7:**
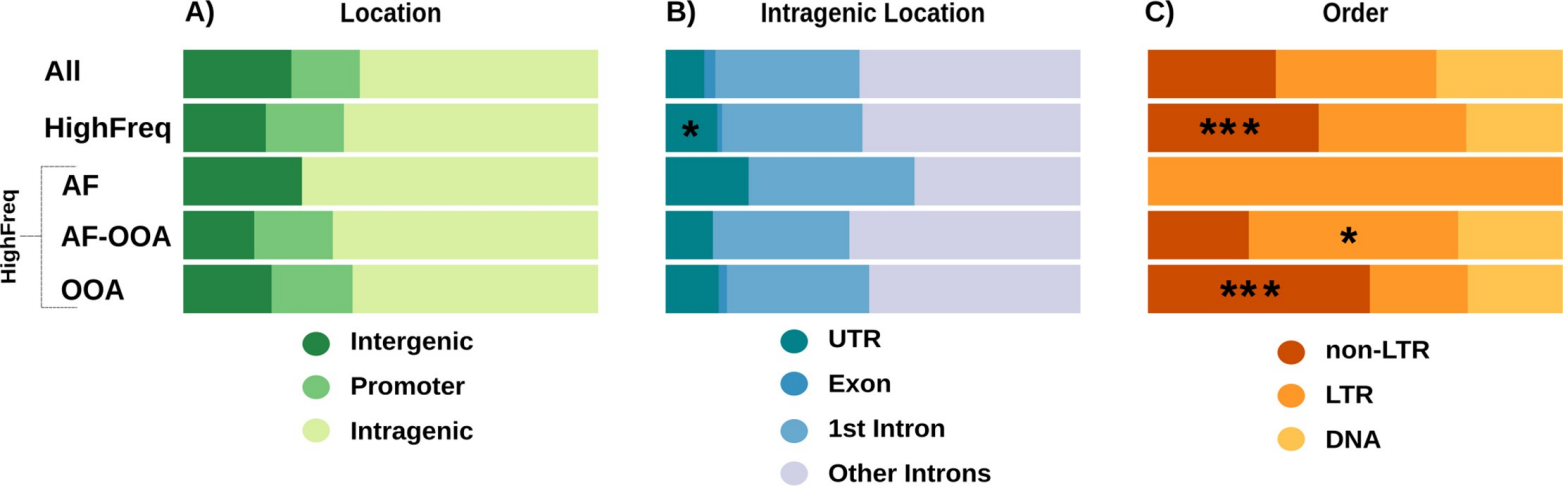
Caracteristics of the HighFreq TEs. **A)** TE location regarding the nearest gene. **B)** Location of intragenic TEs. **C)** TE order. *: p-value < 0.05. ***: p-value < 0.001 (Chi-square test).

We tested whether the genomic location of HighFreq TEs differed from the location of all TEs in the genome. We classified the TEs as present in intergenic, promoter, or genic regions (see Material and Methods). We found no differences between the distributions of HighFreq vs all TEs in the genome (Chi-square test, p-values > 0.05, Fig 7A, S12A Table). Similar results were obtained when we considered the three HighFreq TEs subgroups (S12A Table). We further classified intragenic TEs in exonic, UTRs, 1^st^ intron, and other introns. Only HighFreq TEs were enriched in UTR regions (Chi-square test, p-value < 0.043) (Fig 7B, S12B Table).

We also checked whether the proportion of DNA, LTR, and non-LTR TE orders differed between HighFreq TEs and all TEs in the genome. We found that the HighFreq group contains a larger proportion of non-LTR TEs (42% vs 31%, Chi-square test, p-value = 5.73e-06, Fig 7C, S13 Table). Moreover, when considering HighFreq subgroups we found that OOA TEs also contain a large proportion of non-LTR elements (53% vs 31%, Chi-square test, p-value = 1.79e-11) while the AF-OOA TEs contain more LTR elements (50% vs 39%, Chi-square test, p-value = 1.08e-02) (Fig 7C, S13 Table). Regarding TE families, we found that the HighFreq TEs contain a larger proportion of several families including *jockey*, *297*, *BS* and *pogo* families (S14 Table). When considering only OOA TEs, we found a larger proportion of several families including *jockey*, *F*, and *BS*, while in the AF-OOA there was a larger proportion of *297*, *Quasimodo*, and *opus* (Chi-square test, Bonferroni corrected p-values < 0.05) (S14 Table).

## Discussion

In this work, we identified 300 polymorphic reference TEs present at high frequencies in natural populations, and located in genomic regions with high recombination, where the efficiency of selection is high [35, 40]. Most of these TEs are young insertions suggesting that they have increased in frequency relatively fast (Fig 3). In addition, these insertions are longer compared with other TEs in the genome, also suggesting an adaptive role because long insertions are more likely to act as substrates for ectopic recombination leading to chromosome rearrangements that are often deleterious [16, 34, 43] (Fig 4). Our dataset of 300 putatively adaptive TEs contains all the insertions present at high population frequencies that have previously been identified as putatively adaptive [7, 21, 22, 51–58, 60, 61]. Note that we, and others, have found signatures of positive selection and/or functional evidence for the adaptive role of 55 of the 300 polymorphic adaptive TEs identified in this work, further suggesting that this dataset is enriched for adaptive insertions (Table 2). Besides these 300 polymorphic TEs, we identified 21 fixed reference TEs that are also likely to be adaptive (Table 2). The other 8 TEs that have been previously identifed as candidate adaptive TEs were fixed insertions that did not show signatures of selection or were present at low frequencies in the populations analyzed in this study (Table 2).

Although we looked for evidence of hard and soft sweeps, and for evidence of population differentiation using the F_ST_ statistic, adaptive mutations could show other signatures of selection as well [1, 2, 68]. Polygenic adaptation, which should lead to modest changes in allele frequency at many loci, would be overlooked by the more conventional methods for detecting selection used in this study [69]. A recent work used F_ST_ and gene set enrichment analysis to find evidence of polygenic adaptation in European *D*. *melanogaster* populations [53]. In addition, analysis of environmental correlations between allele frequencies and ecological variables could also lead to the identification of additional TE insertions under positive selection [70–73]. Thus, further analysis could lead to the identification of signatures of selection in other insertions in our dataset besides the 55 polymorphic insertions that showed signatures of selection identified in this work (Table 2).

Our dataset of 300 putatively adaptive polymorphic TEs allowed us investigating global patterns in the biological functions that might be affected by TE-induced adaptive mutations in the *D*. *melanogaster* genome. Previous genome-wide screenings looking for adaptive TE insertions identified a small number of candidates that preclude the identification of the putative traits under selection [7, 8, 21, 22]. In this work, we found that genes nearby putatively adaptive TEs are enriched for response to stimulus, development, and behavioral and learning functions (Fig 6). Through literature searches, we found that 41% (148 out of 363) of these genes have previously been identified as candidate stress-related genes including xenobiotic stress, desiccation, and cold stress (Fig 6). If we focus on the subset of TEs that are likely to be involved in out-of-Africa adaptations, we found similar gene functional enrichments (S6 Fig). Interestingly, circadian behavior gene functions are enriched in this dataset of TEs, consistent with adaptation to seasonal changes in daylight experienced by flies in their out-of-Africa expansion [74]. Thus, our results showed that TE-induced adaptive mutations are mainly likely to contribute to stress-response, developmental, and behavioral traits. Although these traits have previously been identified as targets of natural selection, our results point to the most likely causal variant rather than to a group of linked SNPs [75–77]. Thus, although challenging and time-consuming, follow-up functional analysis of these adaptive mutations should confirm their causal role, as we, and others, have already demonstrated in the past [52, 54–56, 58, 60, 61].

Most of the signatures of positive selection found in the regions flanking the putatively adaptive insertions were continent specific (S3B Fig). These results suggest that a significant proportion of the 300 putatively adaptive TEs could be involved in local adaptation. Thus, it is likely that by exploring more natural populations we could identify additional adaptive insertions. We are also missing TEs that could be playing a role in seasonal and altitudinal adaptation, as both dimensions have been shown to be relevant for *D*. *melanogaster* [78–80]. Finally, our study is also limited to those insertions present in the reference genome. Although there are several packages that infer the presence of *de novo* TE insertions in genome sequencing data, they have a high rate of false positives and none of them provides the precise genomic coordinates of the insertions, which result in inaccurate TE frequency estimations [10, 81]. In addition, the size and the age of the *de novo* insertions cannot be estimated hindering the characterization of putatively adaptive insertions [81, 82]. Long-read sequencing techniques should, in the near future, help overcome this limitation and allow the community to investigate the contribution of non-reference TE insertions to adaptive evolution [83].

We also found that the presence of 19 of the candidate adaptive TEs correlated with changes in expression, both up-regulation and down-regulation, of nearby genes (Table 3 and S11 Table). For four of these TEs, *FBti0018880*, *FBti0019627*, *FBti0019386*, and *FBti0019985*, changes in expression of the nearby genes have also been reported based on allele-specific expression and/or qRT-PCR experiments, and further shown to be associated with changes in fitness-related traits [52, 54, 55, 58, 84]. In addition to these 19 insertions, another four TEs *FBti0020119*, *FBti0020057*, *FBti0018883*, and *FBti0020137* were associated with allele-specific expression changes [84]. Thus, overall, 23 insertions are associated with changes of expression of nearby genes, which at least in four cases lead to changes in fitness-related traits. Note that because 41% of the genes nearby candidate adaptive TEs are candidates for stress-related phenotypes, it could be that changes in expression are only induced by the TEs in response to stress.

Overall, we identified 300 polymorphic and 21 fixed reference TE insertions likely to be involved in adaptive evolution as suggested by their population frequencies, age, size, and presence of signatures of selection in a subset of them. These TEs substantially add to the list of genetic variants likely to play a role in adaptation in *D*. *melanogaster*. Functional profiling of these candidates should help elucidate the molecular mechanisms underlying these mutations, and confirm their adaptive effect on the traits identified.

## Materials and methods

### Dataset

We analyzed available *D*. *melanogaster* genome sequencing datasets from 91 samples collected in 60 natural populations distributed worldwide (Fig 1 and S1 Table). Most samples (83) were generated using pool-sequencing, while the remaining eight samples came from individually sequenced strains. The distribution of populations across continents was: one from Asia, 39 from Europe, 14 from North America, five from Oceania, and one from Africa. The African population was collected in Zambia, the ancestral range of the species [85]. For this work, we only used the 67 Zambian strains without any European admixture [85]. All data was downloaded from the NCBI Sequence Read Archive (SRA) from published projects as of April 2016, and from data available in our laboratory (S1 Table). Note that we attempted to include five more samples in our dataset, but we were unable to estimate TE frequencies in these samples. These samples were from Queensland and Tasmania [75], Winters [86], Vienna [87], and Povoa de Varzim [22].

### Transposable element frequency estimation

To estimate TE population frequencies, we used *T-lex2*, a computational tool that works both with individual genomes and with pooled samples. *T-lex2* combines the genotyping information obtained for each individual genome to calculate the population frequency, while for pooled samples the frequency is directly estimated from the number of reads providing evidence for the presence and for the absence of each insertion [33]. Population frequencies for 34 European populations estimated using *Tlex-2* were obtained from Mateo *et al*.[53] and Kapun *et al*.[48]. We used *T-lex2* [33] to estimate the population frequency in the other 26 available populations (six populations sequenced as individual genomes, and 20 populations sequenced as pooled samples).We first downloaded genomic coordinates of all the annotated TEs (5,416 TEs) from FlyBase r6.04 [88, 89]. 2,234 of the 5,416 TEs belong to the INE family that has been inactive for the past 3–4.6 Myr [90–92], and were discarded. From the 3,182 non-INE TEs, we excluded nested TEs, TEs flanked by other non-INE TEs (100bp on each side of the TE), and TEs that are part of segmental duplications, because *T-lex2* does not provide accurate frequency estimates for these TEs [33]. After these filtering steps we ended up with 1,630 TEs. For 108 of the 1,630 TEs we used the corrected genomic coordinates as described by Fiston-Lavier *et al*. [33]. *T-lex2* parameters were set to default except for read length and the use of paired reads that were specific for each dataset.

For the eight individually-sequenced populations, *T-lex2* was able to calculate frequencies for the 1,630 TEs in most of the strains (S7 Fig). Indeed, we only considered a TE frequency if we had data from at least 9 strains in a given population, as this is the smallest number of strains in a sample (S1 Table). For the 83 samples that were pool-sequenced, we only considered frequencies calculated with 3 to 90 reads. These minimum and maximum thresholds were selected after comparing the distribution of reads in the 48 DrosEU samples to avoid false positives (very low number of reads) or an excess of coverage due to non-unique mapping or spurious reads [48] (S8 Fig). For one population, we have both individually sequenced genomes, and pooled-sequenced genomes. Using data of the individually sequenced population of Stockholm [53] we found a high correlation with the pool-sequenced data of the same population (Pearson correlation coefficient r = 0.98, p-value < 2.2e-16, S9 Fig), which indicates that there is no bias due to the sequencing strategy when calculating the frequencies using *T-lex2*. For most TEs we could estimate frequency in most of the samples (S10 Fig). We only discarded 15 TEs where *T-lex2* estimated frequencies for less than 10 out of the 91 samples, ending up with a dataset of 1,615 TEs.

We considered a TE to be located in high recombination regions when the two available recombination estimations for *D*. *melanogaster* [93, 94] were greater than 0 in the region where the TE is inserted (S2 Table).

### Detecting inversions and correcting TE frequencies

We analyzed the effect of inversions in TE frequency estimations. We focused on the cosmopolitan inversions: In(2L)t, In(2R)Ns, In(3L)P, In(3R)K, In(3R)Mo, In(3R)Payne, and In(3R)C (S15 Table) [79]. 358 TEs are located inside or overlapping with one of these inversions and 36 TEs are located less than 500kb from an inversion breakpoint. For five samples, there is data available on the presence/absence data of inversions: Zambia [85], France [95], North Carolina (DGRP, USA) [96, 97], Italy and Sweden [53]. For all these datasets, we re-estimated TE frequencies for individual samples by removing the strains containing an inversion. We also removed strains where a TE was located 500 kb upstream or downstream of an inversion present in that strain [79]. Removal of strains was done at the TE level using an *in house* python script. As a result, each TE had a different number of supporting strains. The frequencies calculated removing strains with inversions were equivalent to the original ones (Pearson correlation coefficient r = 0.99, p < 2.2e-16, S11 Fig), indicating that the effect of inversions on TE frequency is rather small in our dataset.

### TE age and TE length ratio

We used a phylogeny-based approach to estimate the age of each TE within each family for the 5,416 TEs annotated in the reference genome. The age was estimated as the unique number of substitutions shared between the two closest TEs assuming that they all derived from a common ancestral TE, *i*.*e*. the divergence between closest TEs. Hence, this approach estimates the time since last activity for each TE. Note that activity includes not only transposition but also other genomic TE movements such as the ones caused by duplications.

When the age estimates were calculated, TE annotations were only available for the release 4. Thus, we started by detecting and annotating the TE families and subfamilies in the release 5 of the reference *D*. *melanogaster* genome. We used the *de novo* homology based approach developed in the REPET suite to build a library of TE consensus [98] (https://urgi.versailles.inra.fr/Tools/REPET/). The consensus are proxies of the TE family and subfamily canonical sequences. We then annotated each consensus by blasting them against the TE canonical sequences from the Berkeley Drosophila Genome Project (www.fruitfly.org/). Each TE sequence was then aligned to its set of annotated TE consensus using a global alignment tool from the REPET suite, called RefAlign. The RefAlign launches pairwise alignments avoiding spurious alignments induced by internally deleted TE sequences [32, 99]. All pairwise alignments from the same TE family were re-aligned to generate profiles using ClustalW v2.0.10 [100]. We manually curated each profile: we removed shared substitutions and indels using another tool in the REPET suite called *cleanMultipleAlign*.*py* [32, 99]. A limitation of alignment-based methods is that short TEs could generate misalignments. Hence, to reduce the impact of misalignments 25 TEs shorter than 100bp were removed. For eight TE families (*aurora*, *BS4*, *frogger*, *R1-2*, *Stalker3*, *TART-B*, *TART-C*, and *Xanthias*) composed by less than three copies, we failed to estimate the divergence of the copies and thus were not further considered in this study (11 copies in total). Some profiles were re-aligned using MAFFT v.7 in order to refine conserved regions between TE sequences [101]. For each TE profile, a phylogenetic tree was inferred using the phyML program with the Hasegawa–Kishono–Yano (HKY) model, with different base frequencies. We used the BIONJ technique to build the starting tree and optimized the topology and branch lengths [102]. Finally, the terminal branch lengths were extracted using the Newick Utilities v.1.6 and were used as a proxy for the age of the insertions [103]. We ended up with the age estimates for 5,389 TE sequences from 116 TE families belonging to all TE orders.

We analyzed the length of the TEs by calculating the “TE length ratio (%)” defined as the length of each TE divided by the family canonical length and expressed in percentage. Then, we applied the Wilcoxon rank sum test for determining whether the distribution of the TE Length Ratio values was different between different TE classes.

### Signatures of selective sweeps

In order to detect signatures of positive selection we applied three different methods for identifying selective sweeps: *iHS* [45], *H12* [46], and *nSL* [47]. We separately analyzed two datasets of individually sequenced populations from Europe and North America. For the EU populations we used sequences from 158 strains belonging to four different populations: 16 strains from Castellana Grotte (Bari, South Italy) [53], 27 strains from Stockholm (Sweden) [53], 96 strains from Lyon (France) [95, 104] and 19 strains from Houten (The Netherlands) [105]. We pooled the sequences from the four European populations as it has been described that there is no evidence of latitudinal population structure in European populations [48]. This allowed us to analyse a similar number of strains in the two continents. For the Sweden and Italian populations, we first obtained the *vcf* and *bam* files from [53], we filtered out all non-SNP variants and then we used *Shapeit v2*.*r837* [106] for estimating haplotypes (phasing). For the French and Dutch populations we first downloaded consensus sequences from the Drosophila Genome Nexus (DGN) 1.1 [104], and we then created a *SNP*-*vfc* file using a custom python script. We then merged all EU populations in a single *SNP-vcf* file using *vcftools v*.*0*.*1*.*15* [107]. For the NA population we used the *SNP*-*vcf* file as provided by the Genetic Reference Panel (DGRP) for 141 strains collected in Raleigh, North Carolina [96, 97].

*iHS* was calculated using the *iHSComputer* software (https://github.com/sunthedeep/iHSComputer). We created *iHSComputer* input files (*SNPs-TEs* files) by adding the *T-lex2* information to the *SNP-vcf* file. For each TE and each strain we codified the presence/absence of the TE in a biallelic way and place them in the midpoint coordinate of the TE. Note that only presence/absence results from *T-lex2* were taken into account, leaving “polymorphic” and “no data” as missing data positions [33]. The presence of the TE was considered as the ´derived´ state and the absence as the ´ancestral´ state. Since *iHSComputer* runs for each chromosome separately, we created 100 kb-windows recombination files for each chromosome based on the recombination map from [93]. We standardized iHS values according to Voight *et al*. [45] and determined its significance by comparing iHS value for the TEs against the empirical distribution of iHS values for SNPs falling within the first 8–30 base pairs of small introns (< = 65 bp) which are considered to be neutrally evolving [108]. Two empirical distributions were generated: one for the SNPs present at high frequency in the out-of-Africa and in the African populations, and another one for SNPs present at high frequency in out-of-Africa populations but present at low frequency in the African population (S12 Fig). TEs with *iHS* values falling outside the 5th percentile of the corresponding empirical distribution of neutral SNPs were considered significant.

The *H12* statistic was calculated using the SelectionHapStats software (https://github.com/ngarud/SelectionHapStats/, [46]. We formatted the *SNPs-TEs* files previously used in the *iHS* calculation and run the *H12_H2H1.py* script for each TE in the *singleWindow* mode using 100 SNPs as the window size. We first selected windows in the top 15% most extreme H12 values. We then checked whether haplotypes in these windows contained the TE in at least 50% of the strains for at least one of the three most frequent haplotypes. Only TEs that fulfil this condition were considered significant. Note that 17 out of the 18 significant TEs are present in the first or second most frequent haplotype.

The *nSL* statistic was calculated using *selscan v1*.*1* [109]. Input files were generated based on the *SNPs-TEs* files from the *iHS* calculation. We created one *tped* file for each TE and removed all strains and positions containing missing data. Extreme *nSL* values were determined using the *norm* program for the analysis of *selscan* output. Unstandardized *nSL* values were normalized in 10 frequency bins across the entire chromosome and significant *nSL* values were determined using the—*crit-percent* 0.05 parameter.

### Population differentiation using F_ST_ for latitudinal distant populations

We calculate the Fixation index (F_ST_) between pairs of latitudinal distant populations for each of the three continents. We created *vcf* files for the TEs based on *T-lex2* results and used *vcftools v*.*0*.*1*.*15* [107] for calculating the pairwise F_ST_ estimator [49]. The pairwise calculations performed for each continent were: Europe: Italy vs Sweden [53] and Vesanto vs Nicosia [48]; Oceania: Innisfail vs Yering [110] and Queensland vs Tasmania [77] and North America: Maine vs Florida [77], and Maine vs Florida [78]. For each pair, we calculated F_ST_ values for all TEs and tested them against the empirical distribution of F_ST_ values of neutral SNPs while controlling for TE frequency in the African population [85]. Innisfail, Yering, Maine and Florida SNP callings were obtained from the Dryad Digital Repository (http://datadryad.org/resource/doi:10.5061/dryad.7440s, [78, 110]. Queensland, Tasmania, Florida and Maine SNP callings from Reinhardt *et al*. [77] were provided by Dr. Andrew Kern. Italy and Sweden SNP callings were obtained from Mateo *et al*. [53]. Vesanto and Nicosia SNP callings were obtained from Kapun et al. [48]. F_ST_ values for neutral SNPs were also calculated using *vcftools v*.*0*.*1*.*15* [107]. Then, for each pairwise comparison we created two empirical distributions of F_ST_ values of neutral SNPs: one for SNPs that were at low frequency in Zambia and other for SNPs that were at high frequency in Zambia. F_ST_ values of TEs at high frequency in Zambia were compared with the distribution of neutral SNPs F_ST_ at high frequency in Zambia and F_ST_ values of TEs at low frequency in Zambia were compared with the low frequency SNPs distribution. We considered a TE to be significantly differentiated when its F_ST_ value was greater than the percentile 95th of the corresponding empirical distribution.

Overall, we calculated F_ST_ values for 254 TEs in at least one pair of populations and we found 78 of them showing extreme values when compared with the distribution of F_ST_ from neutral SNPs (S6 Table). 67 of these 78 TEs were consistently present at high frequencies in populations located in high latitudes or in low latitudes. 43 of the 67 TEs were present at high frequencies in low latitude populations in at least one pairwise comparison, and 24 TEs were present at high frequencies in high latitude populations in at least one pairwise comparison (S6 and S16 Tables). Finally, to be conservative, we only considered those TEs with significant F_ST_ values in at least two populations and always present at high frequencies in populations located in high or low latitude (concordant F_ST_).

### Evidence of selection for fixed TEs

Following Kofler *et al*. (2012) [22], we investigated whether Fixed TEs showed evidence of selection. In order to do that, we calculated Tajima´s D values [50] in non-overlapping 500 bp window using *vcftools v*.*0*.*1*.*15* [107]. We identified windows showing significant low Tajima´s D values by selecting windows with values lower than the 5% quantile of the whole genome distribution, matching by recombination rate and type of chromosome. Cutting values were -1.65 for the autosomes and -1.82 for the X chromosome. We then looked whether significant windows were overlapping or nearby Fixed TEs boundaries.

### TE location

We analyzed whether TEs were located at specific regions in the genome regarding the nearest gene. We used TEs and gene coordinates from FlyBase r6.04 [88, 89] and considered both coding and non-coding genes. For each TE, we determined whether it was located inside a gene or in an intergenic region. We further classify the TEs located in intergenic regions in those located at more or less than 1kb of the nearest gene. For TEs present inside a gene we further determined the class site overlapping with the TE annotation: *Exon*, *UTR*, *Intron*. If the TE is inserted in an intron, we checked whether it was inserted in the first intron, where is more likely to affect expression [111, 112].

### Expression quantitative trait loci (eQTL) analysis

We use *Matrix eQTL* v2.1.1 [64] to calculate correlations between the presence/absence of the TEs and the expression of nearby genes. We used expression data from the DRGP lines (Raleigh, North Carolina, [65]) as available in the DGRP2 repository (http://dgrp2.gnets.ncsu.edu/data.html) and the presence/absence TE information for the DGRP lines for which *T-lex2* was successfully run (see above). *T-lex2* identified 1,603 TEs in the DRGP lines and 1,177 of them contain at least one gene at less than 1kb of any of the two junction coordinates of the TEs. One line (RAL-591) was not present in the expression data, so we ended up with 140 lines in the dataset. For each line, we used the average of the normalized gene expression value from the two replicates and analyzed female and male data separately. For the genotyping data, we used both the start and the end coordinates of the 1,615 TEs as positions in the genome and codified the absence (0), polymorphic (1), presence (2) and no data (NA) from *T-lex2* output using a custom python script. *Matrix eQTL* was run with default parameters, applying only the *Linear* model and with a *cisDist = 1000*, meaning that we considered only genes that were at less than 1kb from any of the junction coordinates of the TE. We then evaluated the significance of the correlations as provided by the *Matrix eQTL* software and we considered TEs that were significant in at least one sex. From the 1,177 analyzed TEs, we kept only the 638 TEs located at high recombination rate regions and classified them according to their frequency in the DGRP population as: HighFreq (10% < frequency < 95%), LowFreq (frequency ≤ 10%) and Fixed (frequency ≥ 95%). LowFreq TEs were further classified as Private if only one strain was containing the TE. 235 of the 300 candidate adaptive TEs were included in the 638 dataset.

### Functional enrichment analysis

We performed functional enrichment analysis for Gene Ontology (GO) biological process for the genes nearby TEs using the DAVID functional annotation cluster tool (*v*.*6*.*8*) [62, 63]. Based on TEs and genes coordinates from FlyBase r6.04 [88, 89], we selected genes located at less than 1kb as the ones putatively likely affected by the TEs, since this is the approximate size of the promoter region in *D*. *melanogaster* [113]. If there were no genes at less than 1kb, we selected the closest one. All comparisons were performed using the full list of genes in *D*. *melanogaster* as the background. We considered DAVID clusters as significant when the enrichment score (ES) was higher than 1.3 as described in Huang da *et al*. [62].

Our dataset of 300 HighFreq TEs is enriched for insertions belonging to 18 TE families (S14 Table). We thus tested whether TEs belonging to these families and located in high recombination regions were located nearby genes enriched for particular GO biological processes. We found that three of these families, *297*, *jockey*, and *transib2*, where located nearby genes enriched for several biological processes (S8E Table). We thus removed the TEs belonging to these three families from our 300 HighFreq dataset and re-run DAVID. We found that the GO enrichments did not significantly change when we removed the insertions from these three families (S8F Table).

In addition, in December 2016 we searched the literature using PubMed to find publications that identified genes associated with phenotypic traits studied in the DGRP project (olfactory behavior, alcohol exposure, desiccation, aggressiveness, cold tolerance, pigmentation, starvation, mating behavior, and oxidative stress). We also included phenotypic traits for which there is gene expression data available (heavy-metal stress, xenobiotic stress, diapause, locomotor behavior, and hypoxia). Finally, we looked for publications related with immunity, heat-shock stress, and circadian behavior as these three are relevant adaptive traits in Drosophila. We included genome-wide studies (GWAS, QTL, gene expression, and protein-protein interactions) and candidate-gene studies (S9 Table). We generated lists of candidate genes for each one of the 17 different fitness-related traits. We then converted the gene names to Flybase gene identifiers. This step was necessary because in *D*. *melanogaster* genes often have more than one name but all genes have a single Flybase identifier. To construct our final candidate gene lists, we only considered those genes that were present in two or more independent publications. We then checked whether the genes nearby the 300 HighFreq TEs, the 84 TEs with evidence of positive selection, the 174 OOA, and the 111 AF-OOA TEs were present in our candidate gene lists. We used the hypergeometic test to determine whether different sets of TEs showed more genes previously associated with different stress-related and behavior-related traits than expected by chance.

## Supporting information

S1 Fig Distribution of number of TEs that are present at >0.10 and < 0.95 frequency by number of populations in which they are present at that frequency. We considered TEs to be present at high frequency (HighFreq) when they fulfil the frequency condition in at least three samples (represented by blue bars in the figure).
(PDF)

S2 Fig Comparison of age estimations obtained by Bergman and Bensasson (2007) and the estimations obtained in this work. Only the 417 TEs that are common between the two studies are plotted. A) TE age distribution of the 417 TEs based on Bergman and Bensasson (2007) and in this work. Note that there are 10 insertions that showed extreme age values in our dataset (> 0.12). B) Correlation between the two age estimates before and after removing the 10 TEs with extreme age values in our data set (n = 407).
(PDF)

S3 Fig **Boxplots showing the distribution of TE ratio percentages** (percentage of the length of the TE insertion regarding the length of the canonical family sequence) for each TE category and colored by Age (A) and TE class (B).
(PDF)

S4 Fig Venn diagrams for the 36 HighFreq TEs with significant evidence of selective sweeps. **A)** Overlapping between TEs showing significant results for the different selective sweeps statistics (*iHS*, *H12* and *nSL*)**. B**) Overlapping between TEs showing at least one significant test in the North American (NA) and/or the European (EU) population. The percentage between brackets is regarding the total number of significant TEs (36). Numbers between square brackets show the number of TEs for which we were able to calculate at least one of the sweep statistics.
(PDF)

S5 Fig Venn diagrams showing the overlap between TEs showing significant F_ST_ values in at least one pair of populations. A) TEs present at high frequency in populations located at low latitude locations. B) TEs present at high frequency in populations located at high latitude locations.
(PDF)

S6 Fig Functional enrichment analysis of genes nearby OOA and AF-OOA TEs. **A) Significant Gene Ontology Clusters according to DAVID functional annotation tool.** Only the top six significant clusters are showed (enrichment score > 1.3). The horizontal axis represents DAVID enrichment score (see S9C and S9D Table for details). **B)** Significantly overrepresented fitness-related genes according to previous genome association studies. All FDR corrected p-values < 0.05, Chi-square test (see S11C and S11D Table for details). The horizontal axis represent the log_10_(χ^2^). In both cases, A) and B), numbers nearby each bar indicate total number of genes in that category. Bar colors indicate similar biological functions of the clusters (**A**) and the fitness-related traits (**B**): green: stress response; red: behavior; blue: development; and yellow: transport.
(PDF)

S7 Fig Distribution of the number of TEs (y axis) by the number of strains for which *T-lex2* estimated frequencies in the 8 individually-sequenced populations. (PDF)

S8 Fig Distribution of mapped reads for the presence module (red), absence module (green) and total number of reads (blue) for each one of the 48 DrosEU samples (Kapun *et al*. 2018). (PDF)

S9 Fig Correlation between frequencies estimated with data obtained using different sequencing strategies in the Stockholm (Sweden) population. Frequencies calculated using individual strain sequencing (x) (Mateo et al 2018) and pool sequencing (y). Pearson correlation coefficient r = 0.98, p-value < 2.2e-16.
(PDF)

S10 Fig Histogram showing the number of TEs (y axis) and the number of samples for which we were able to estimate its frequency. (PDF)

S11 Fig TE frequencies estimated using all strains (x axis) vs frequencies estimated after removing strains that contain inversions (y axis) for different individually-sequenced populations. **A)** Zambia (Lack et al., 2015), **B)** France (Pool et al., 2012), **C)** DGRP (Raleigh) (Huang *et al*. 2014; Mackay *et al*. 2012), **D)** Italy (Bari) and **E)** Sweden (Stockholm) (Mateo et al 2018). All Pearson correlation coefficients r = 0.99 and p-value < 2.2e-16.
(PDF)

S12 Fig Distribution of iHS values obtained for TEs (red) and neutral SNPs (cyan) in the North American population (DGRP, Raleigh, North Carolina). **A)** Distribution of iHS values for all TEs and neutral SNPs. **B)** Distribution of iHS values for TEs and neutral SNPs at high frequency (> 0.10) in the OOA population (Raleigh) and in the African population (Zambia). **C)** Distribution of iHS values for TEs and neutral SNPs at high frequency (> 0.10) in the OOA population, but at low frequency in the African population.
(PDF)

S1 Table Information for the 91 samples used in this study. (XLSX)

S2 Table Frequency estimations using Tlex2 for the 1,615 TEs at each of the 91 samples. NA indicates that the frequency could not be estimated for that TE in the given sample. Recombination estimates according to Comeron *et al*. (2012) and Fiston-Lavier *et al*. (2010) are showed for each TE. Class column indicates the category at which each TE was classified.
(XLSX)

S3 Table TE length ratio statistics. At the top, mean and median TE Length Ratio (%) for each TE category. At the bottom, results for the Wilcoxon rank sum test and Kruskal Wallis test among different TE categories. The results are shown for All TEs (A), for onlt young TEs (B), for only old TEs (C), for only TEs of the class DNA (D), for only TEs of the class LTR (E) and for only TEs of the class non-LTR (F).
(XLSX)

S4 Table Number of TEs showing significant values in the selection tests for each HighFreq category. For each sweep test (*iHS*, *H12* and *nSL*), “Continent” column indicates population used for the analysis: NA: North America or EU: Europe. For each HighFreq category, table shows the number of significant TEs / number of TEs for which the test was calculated. “At least one test” indicates the number of TEs at each category showing at least one test significant / TEs with at least one test calculated.
(XLSX)

S5 Table List of 36 TEs showing at least one significant (highlighted in red) selective sweep test (*iHS*, *H12* or *nSL*). (XLSX)

S6 Table List of the 254 HighFreq TEs with at least one pairwise F_ST_ calculation performed. Category indicates the classification of the TE according to Fig 2. For each continent, two pairwise comparisons were performed. Values for each comparison are the F_**ST**_ (in red the significant ones). Concordant F_**ST**_ indicates whether TEs with significant F_**ST**_ were at high frequency in the same climate zone in more than one population. Concordance information indicates, for each significant pairwise calculation (separated by ´;´) the continent (EU, NA or OC) and the climate zone at which the TE is a higher frequency (Tropical/Mild Temperature, Snow).
(XLSX)

S7 Table Summary results for Fixed TEs showing significant Tajima´s D values on neighbour windows. Significance was dermined by the 5% quantile of Tajima´s D values from all high recombination regions in the genome (-1.65 for Autosomes and -1.82 for chromosome X).
(XLSX)

S8 Table **A** Results of gene ontology (GO) enrichment test for the 83 genes nearby the 65 TEs showing evidence of selection (ES). **B Table**. Results of gene ontology (GO) enrichment test for the 363 genes nearby the 300 HigFreq TEs. **C Table:** Results of gene ontology (GO) enrichment test for the 215 genes nearby the 174 OOA TEs. **D Table.** Results of gene ontology (GO) enrichment test for the 143 genes nearby the 111 AF-OOA TEs. **E Table**. Results of gene ontology (GO) enrichment test for genes nearby TE insertions belonging to the *297, jockey, and transib2* families. **F. Table**. Results of gene ontology (GO) enrichment test for genes nearby the 300 HighFreq TEs, but excluding TEs from the *297, jockey*, and *transib2* families.
(XLSX)

S9 Table Gene association studies analyzing different fitness-related phenotypes. (XLSX)

S10 Table Enrichment of genes previously described as associated with different stress-related and behaviour-related traits in the different datasets analyzed. A) Genes the 65 TEs with evidence of selection. B) Genes nearby the 300 HighFreq TEs. C) Genes nearby the 174 OOA TEs. D) Genes nearby the 111 AF-OOA TEs.
(XLSX)

S11 Table 19 TEs showing significant correlation with the expression of nearby genes. Results are divided in correlations obtained with male and female expression data (Huang et al. 2015). beta: Effect size estimate, t-stat: Test statistic (t-statistic of T-test), p-value: p-value for the linear regression. FDR: False discovery rate estimated with Benjamini–Hochberg procedure. * TEs showing evidence of selection (Table 2, main text).
(XLSX)

S12 Table Genomic location of different TE categories. Percentages and rigth-tail p-values are showed when the Chi-square test is significant. (A) Localization of TEs regarding the nearest gene across categories. (B) Localization of intragenic TEs across TE categories.
(XLSX)

S13 Table TE classes across different TE categories. P-values and percentages are showed in bold when significant enrichment according to Chi-square test p-value < 0.05 when comparing with All TEs.
(XLSX)

S14 Table Enrichment test for TE families. For each family, table shows the number of TEs at each category. HighFreq TEs correspond to the sum of AF, AF-NA, AF-OOA and OOA. p-value (Bonf.) indicates Bonferroni corrected p-values for Chi-square test when comparing HighFreq, AF-OOA and OOA TEs against All TEs. In red p-values < 0.05.
(XLSX)

S15 Table Genomic coordinates of cosmopolitan inversion (Kapun *et al*. 2016) analyzed in order to determine its influence on the transposable elements frequency calculation. (XLSX)

S16 Table Summary statistics for the pairwise F_ST_ calculations. TEs with F_**ST**_: Number of TEs for which it was possible to calculate F_**ST**_. Signif. (Africa H/L): Total number of significant TEs. Between brackets: H: Number of significant TEs identified using the distribution of neutral SNPs that are at high frequency in Africa. L: Number of significant TEs identified using the distribution of neutral SNPs that are at low frequency in Africa (see Material and Methods). Low Latitude (HighFreq): Significant TEs that are at high recombination rate regions (HRR) and are at high frequency only in populations located in low latutidinal regions. High Latitude (HighFreq): Significant TEs that are at high recombination rate regions (HRR) and are at high frequency in populations located in high latutidinal regions. Both (HighFreq): Significant TEs that are at high recombination rate regions (HRR) and are at high frequency in populations from both, low and high latutidinal regions.
(XLSX)
