# Supplementary material for: Stress response, behavior, and development are shaped by transposable element-induced mutations in Drosophila
Source: PLoS Genet. 2019 Feb 12;15(2):e1007900. doi: 10.1371/journal.pgen.1007900 (PMC6372155; doi:10.1371/journal.pgen.1007900)
Supplement: S1 Fig — We considered TEs to be present at high frequency (HighFreq) when they fulfil the frequency condition in at least three samples (represented by blue bars in the figure). (PDF) [file pgen.1007900.s001.pdf]

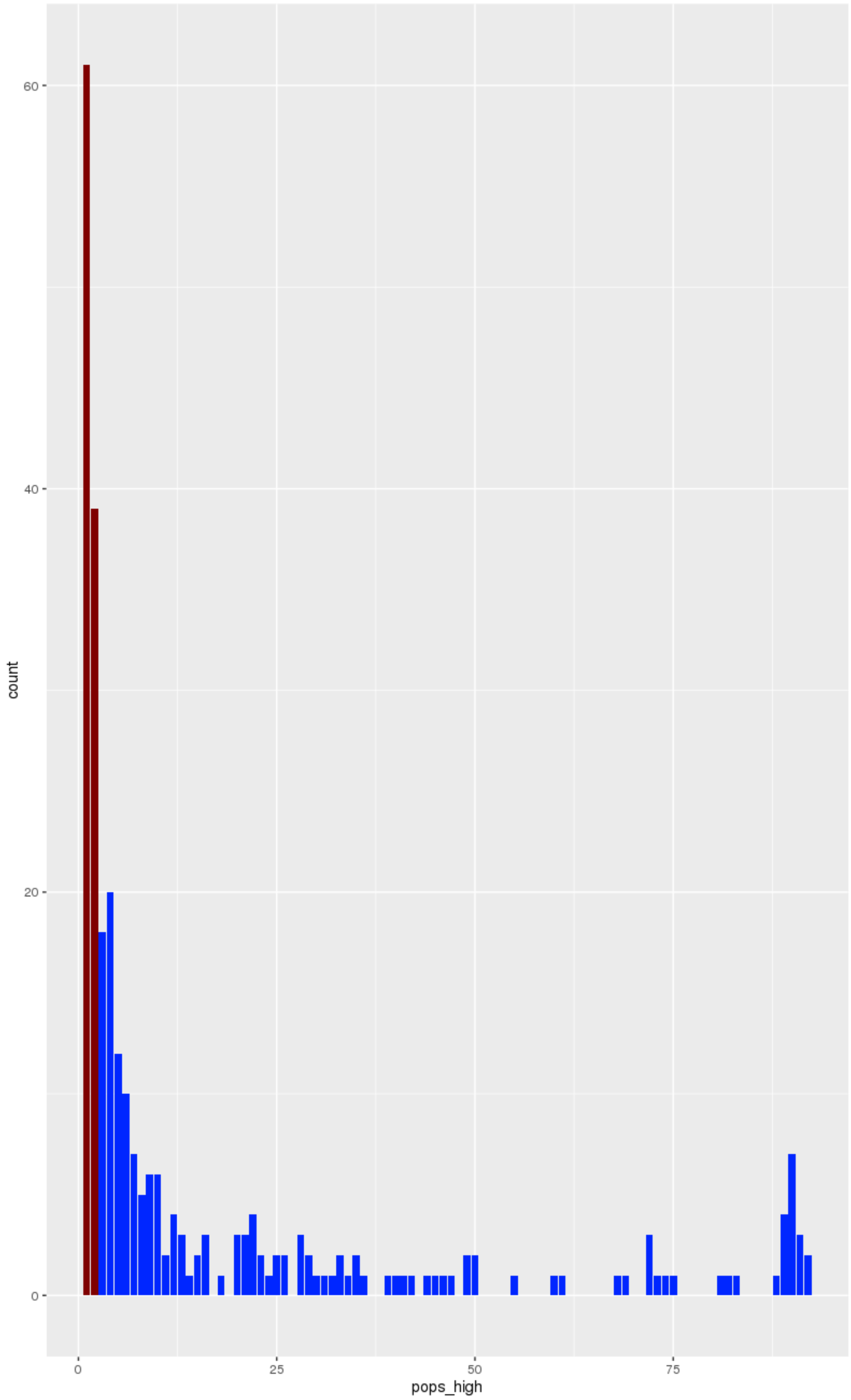

S1 Fig. Distribution of number of TEs that are present at >0.10 and < 0.95 frequency by number of populations in which they are present at that frequency. We considered TEs to be present at high frequency (HighFreq) when they fulfil the frequency condition in at least three samples (represented by blue bars in the figure).
