# Supplementary material for: Stress response, behavior, and development are shaped by transposable element-induced mutations in Drosophila
Source: PLoS Genet. 2019 Feb 12;15(2):e1007900. doi: 10.1371/journal.pgen.1007900 (PMC6372155; doi:10.1371/journal.pgen.1007900)
Supplement: S2 Fig — Only the 417 TEs that are common between the two studies are plotted. A) TE age distribution of the 417 TEs based on Bergman and Bensasson (2007) and in this work. Note that there are 10 insertions that showed extreme age values in our dataset (> 0.12). B) Correlation between the two age estimates before and after removing the 10 TEs with extreme age values in our data set (n = 407). (PDF) [file pgen.1007900.s002.pdf]

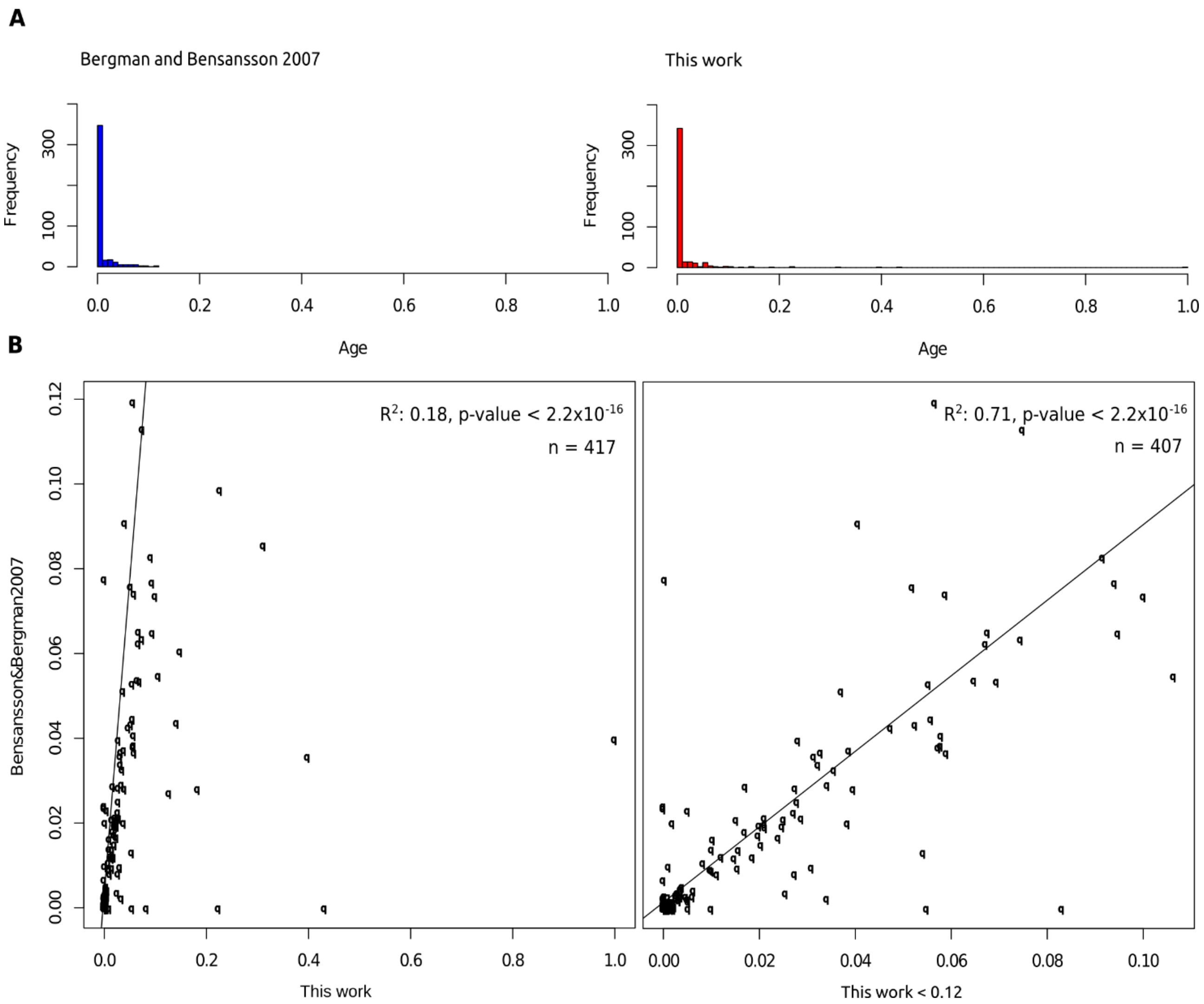

S2 Fig. Comparison of age estimations obtained by Bergman and Bensasson (2007) and the estimations obtained in this work. Only the 417 TEs that are common between the two studies are plotted. A) TE age distribution of the 417 TEs based on Bergman and Bensasson (2007) and in this work. Note that there are 10 insertions that showed extreme age values in our dataset (> 0.12). B) Correlation between the two age estimates before and after removing the 10 TEs with extreme age values in our data set (n = 407).
