# Supplementary material for: Stress response, behavior, and development are shaped by transposable element-induced mutations in Drosophila
Source: PLoS Genet. 2019 Feb 12;15(2):e1007900. doi: 10.1371/journal.pgen.1007900 (PMC6372155; doi:10.1371/journal.pgen.1007900)
Supplement: S3 Fig — Boxplots showing the distribution of TE ratio percentages (percentage of the length of the TE insertion regarding the length of the canonical family sequence) for each TE category and colored by Age (A) and TE class (B). (PDF) [file pgen.1007900.s003.pdf]

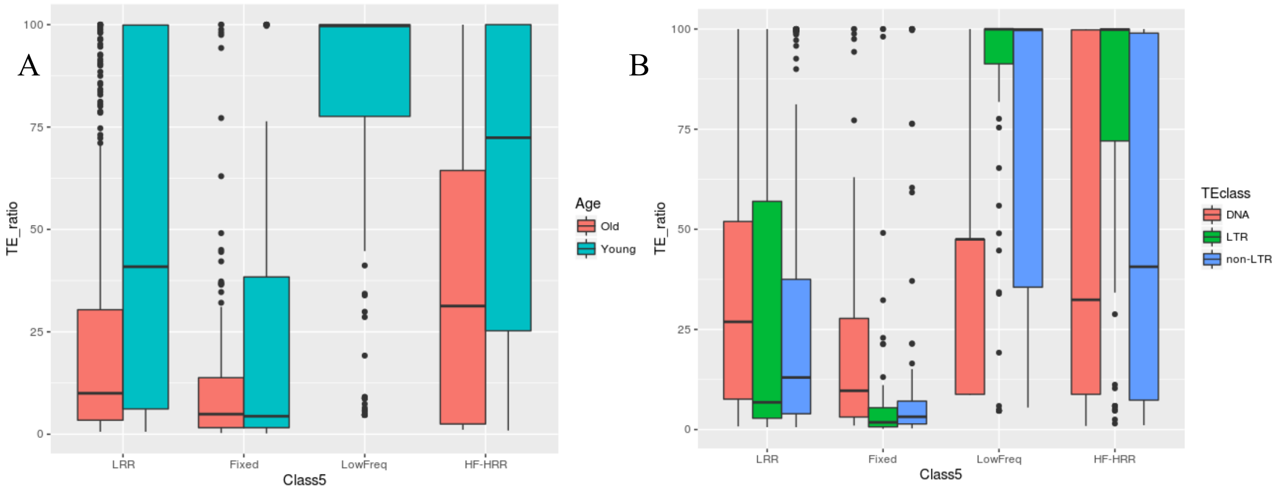

S3 Fig. Boxplots showing the distribution of TE ratio percentages (percentage of the length of the TE insertion regarding the length of the canonical family sequence) for each TE category and colored by Age (A) and TE class (B).
