# Supplementary material for: Stress response, behavior, and development are shaped by transposable element-induced mutations in Drosophila
Source: PLoS Genet. 2019 Feb 12;15(2):e1007900. doi: 10.1371/journal.pgen.1007900 (PMC6372155; doi:10.1371/journal.pgen.1007900)
Supplement: S4 Fig — A) Overlapping between TEs showing significant results for the different selective sweeps statistics (iHS, H12 and nSL). B) Overlapping between TEs showing at least one significant test in the North American (NA) and/or the European (EU) population. The percentage between brackets is regarding the total number of significant TEs (36). Numbers between square brackets show the number of TEs for which we were able to calculate at least one of the sweep statistics. (PDF) [file pgen.1007900.s004.pdf]

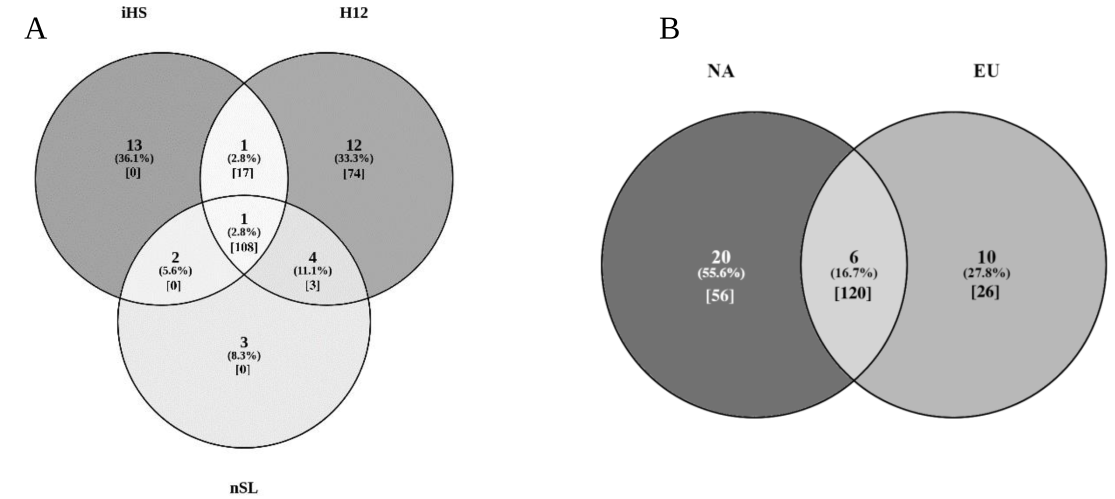

S4 Fig. **Venn diagrams for the 36 HighFreq TEs with significant evidence of selective sweeps.** **A)** Overlapping between TEs showing significant results for the different selective sweeps statistics (*iHS*, *H12* and *nSL*). **B**) Overlapping between TEs showing at least one significant test in the North American (NA) and/or the European (EU) population. The percentage between brackets is regarding the total number of significant TEs (36). Numbers between square brackets show the number of TEs for which we were able to calculate at least one of the sweep statistics.
