# Supplementary material for: Stress response, behavior, and development are shaped by transposable element-induced mutations in Drosophila
Source: PLoS Genet. 2019 Feb 12;15(2):e1007900. doi: 10.1371/journal.pgen.1007900 (PMC6372155; doi:10.1371/journal.pgen.1007900)
Supplement: S5 Fig — A) TEs present at high frequency in populations located at low latitude locations. B) TEs present at high frequency in populations located at high latitude locations. (PDF) [file pgen.1007900.s005.pdf]

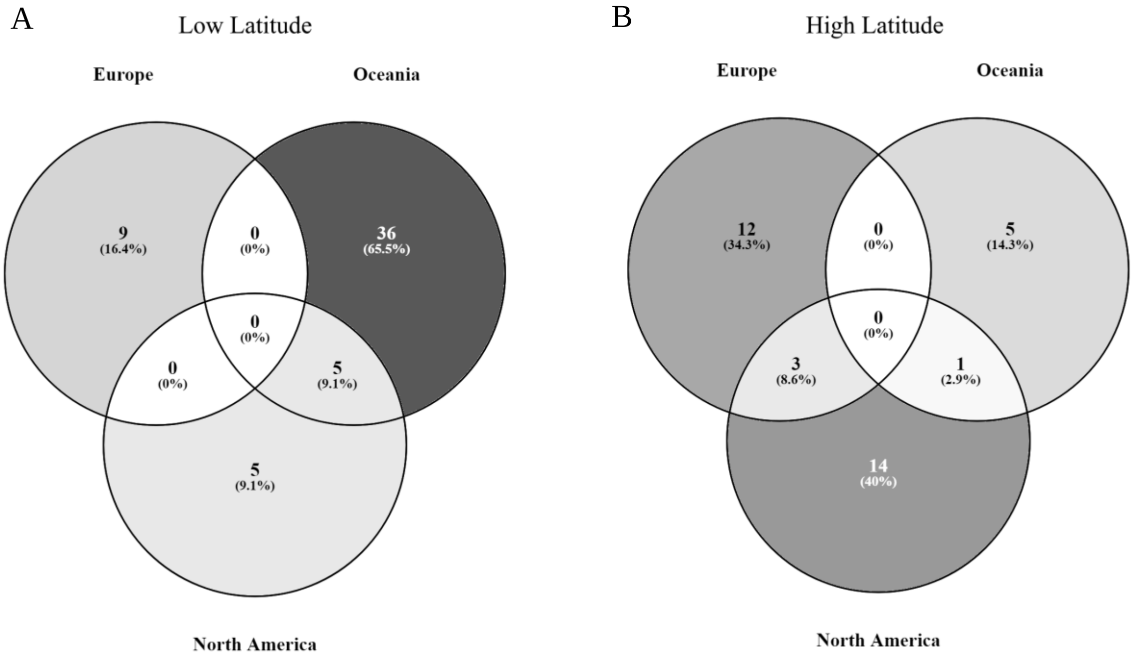

S5 Fig. Venn diagrams showing the overlap between TEs showing significant F_{ST} values in at least one pair of populations. A) TEs present at high frequency in populations located at low latitude locations. B) TEs present at high frequency in populations located at high latitude locations.
