# Supplementary material for: Stress response, behavior, and development are shaped by transposable element-induced mutations in Drosophila
Source: PLoS Genet. 2019 Feb 12;15(2):e1007900. doi: 10.1371/journal.pgen.1007900 (PMC6372155; doi:10.1371/journal.pgen.1007900)
Supplement: S6 Fig — A) Significant Gene Ontology Clusters according to DAVID functional annotation tool. Only the top six significant clusters are showed (enrichment score > 1.3). The horizontal axis represents DAVID enrichment score (see S9C and S9D Table for details). B) Significantly overrepresented fitness-related genes according to previous genome association studies. All FDR corrected p-values < 0.05, Chi-square test (see S11C and S11D Table for details). The horizontal axis represent the log10(χ2). In both cases, A) and B), numbers nearby each bar indicate total number of genes in that category. Bar colors indicate similar biological functions of the clusters (A) and the fitness-related traits (B): green: stress response; red: behavior; blue: development; and yellow: transport. (PDF) [file pgen.1007900.s006.pdf]

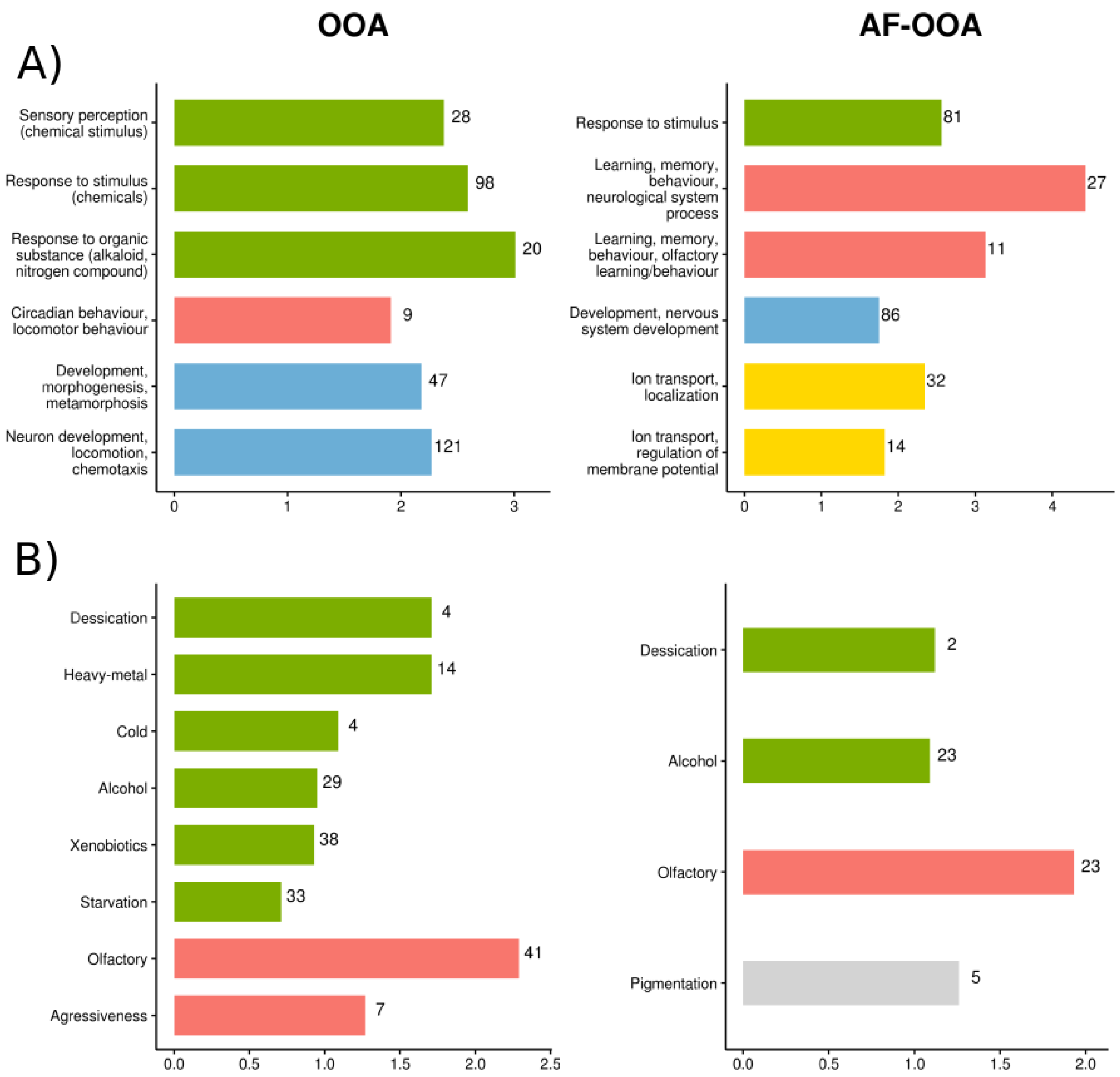

S6 Fig. **Functional enrichment analysis of genes nearby OOA and AF-OOA TEs.** A) Significant Gene Ontology Clusters according to DAVID functional annotation tool. Only the top six significant clusters are showed (enrichment score > 1.3). The horizontal axis represents DAVID enrichment score (see Table S9C and S9D for details). **B)** Significantly overrepresented fitness-related genes according to previous genome association studies. All FDR corrected p-values < 0.05, Chi-square test (see Table S11C and S11D for details). The horizontal axis represent the $\log_{10}(\chi^2)$. In both cases, A) and B), numbers nearby each bar indicate total number of genes in that category. Bar colors indicates similar biological functions of the clusters (**A**) and the fitness-related traits (**B**): green: stress response; red: behavior; blue: development; and yellow: transport.
