# Supplementary material for: Stress response, behavior, and development are shaped by transposable element-induced mutations in Drosophila
Source: PLoS Genet. 2019 Feb 12;15(2):e1007900. doi: 10.1371/journal.pgen.1007900 (PMC6372155; doi:10.1371/journal.pgen.1007900)
Supplement: S7 Fig — (PDF) [file pgen.1007900.s007.pdf]

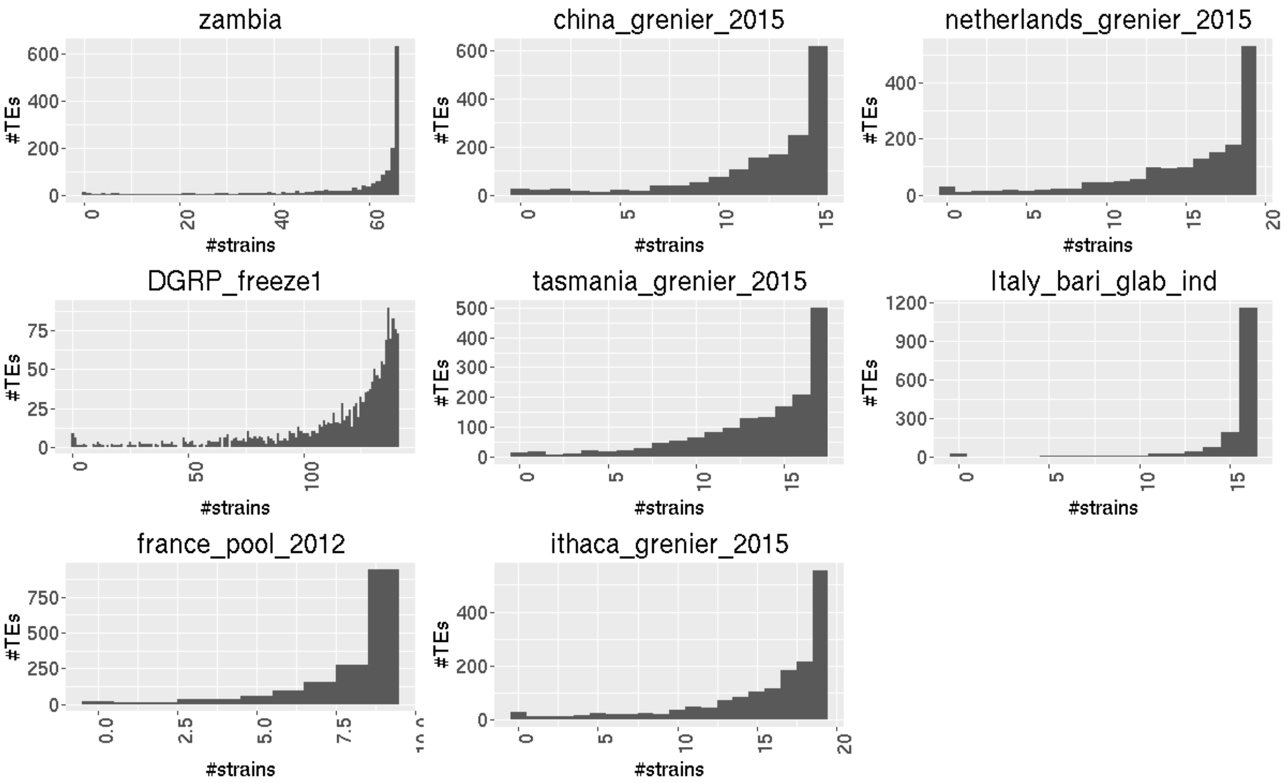

S7 Fig. Distribution of the number of TEs (y axis) by the number of strains for which *T-lex2* estimated frequencies in the 8 individually-sequenced populations.
