# Supplementary material for: Stress response, behavior, and development are shaped by transposable element-induced mutations in Drosophila
Source: PLoS Genet. 2019 Feb 12;15(2):e1007900. doi: 10.1371/journal.pgen.1007900 (PMC6372155; doi:10.1371/journal.pgen.1007900)
Supplement: S8 Fig — (PDF) [file pgen.1007900.s008.pdf]

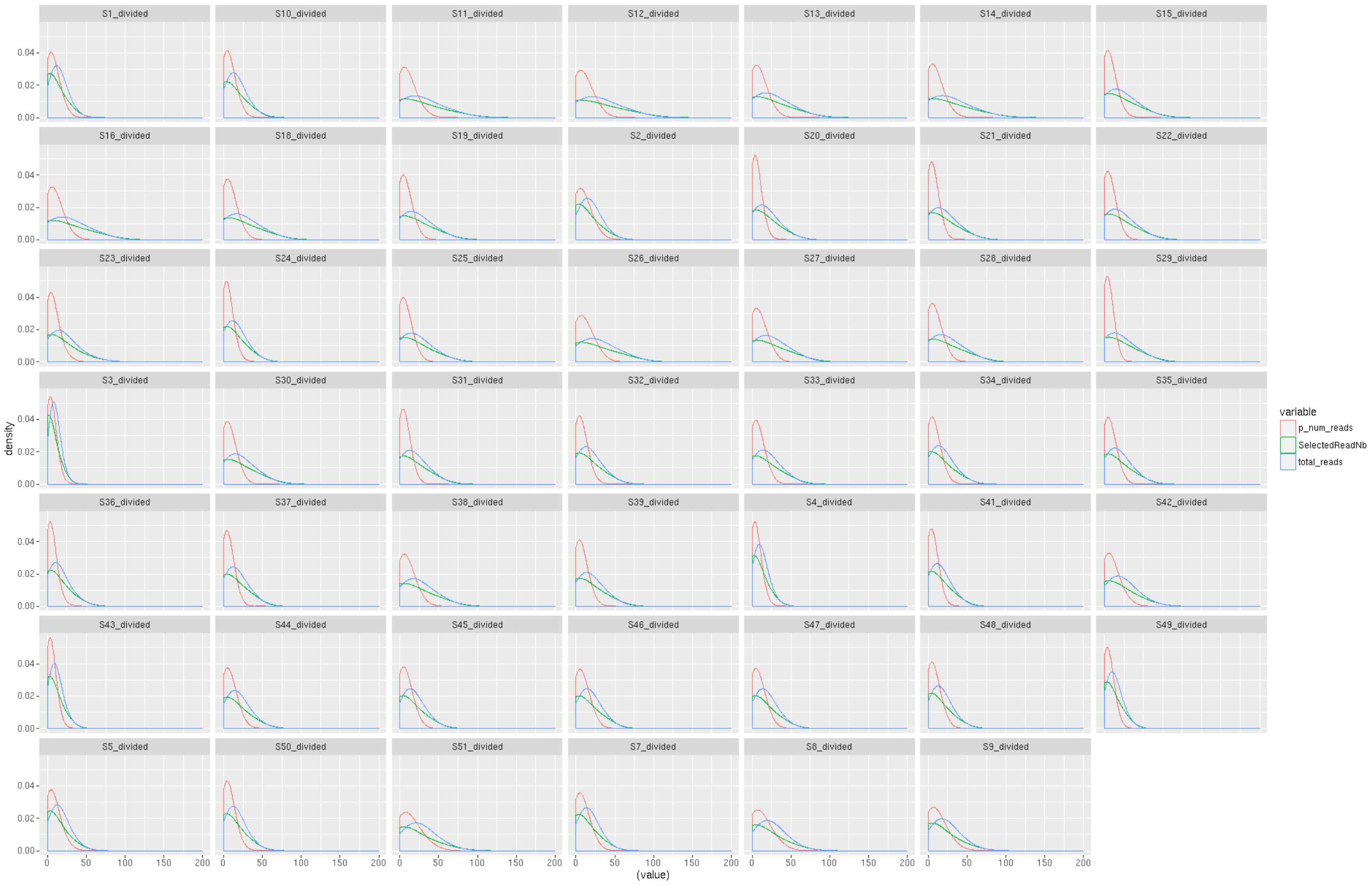

S8 Fig. Distribution of mapped reads for the presence module (red), absence module (green) and total number of reads (blue) for each one of the 48 DrosEU samples (Kapun *et al.* 2018).
