# Supplementary material for: Stress response, behavior, and development are shaped by transposable element-induced mutations in Drosophila
Source: PLoS Genet. 2019 Feb 12;15(2):e1007900. doi: 10.1371/journal.pgen.1007900 (PMC6372155; doi:10.1371/journal.pgen.1007900)
Supplement: S9 Fig — Frequencies calculated using individual strain sequencing (x) (Mateo et al 2018) and pool sequencing (y). Pearson correlation coefficient r = 0.98, p-value < 2.2e-16. (PDF) [file pgen.1007900.s009.pdf]

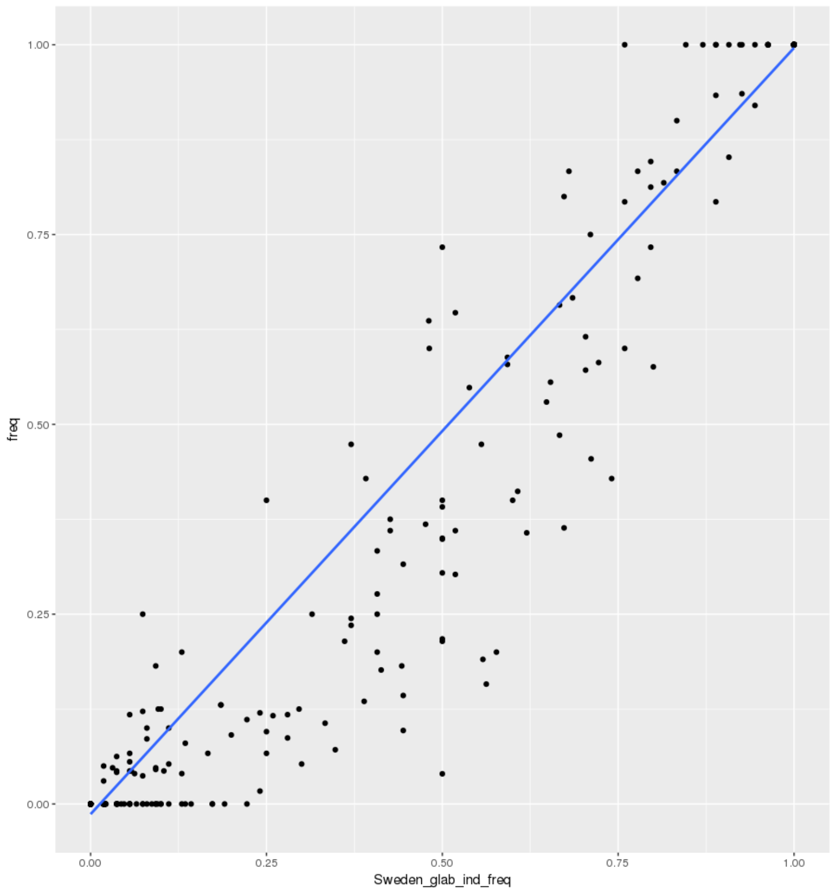

S9 Fig. **Correlation between frequencies estimated with data obtained using different sequencing strategies in the Stockholm (Sweden) population**. Frequencies calculated using individual strain sequencing (x) (Mateo et al 2018) and pool sequencing (y). Pearson correlation coefficient r = 0.98, p-value < 2.2e^{-16}.
