# Supplementary material for: Stress response, behavior, and development are shaped by transposable element-induced mutations in Drosophila
Source: PLoS Genet. 2019 Feb 12;15(2):e1007900. doi: 10.1371/journal.pgen.1007900 (PMC6372155; doi:10.1371/journal.pgen.1007900)
Supplement: S10 Fig — (PDF) [file pgen.1007900.s010.pdf]

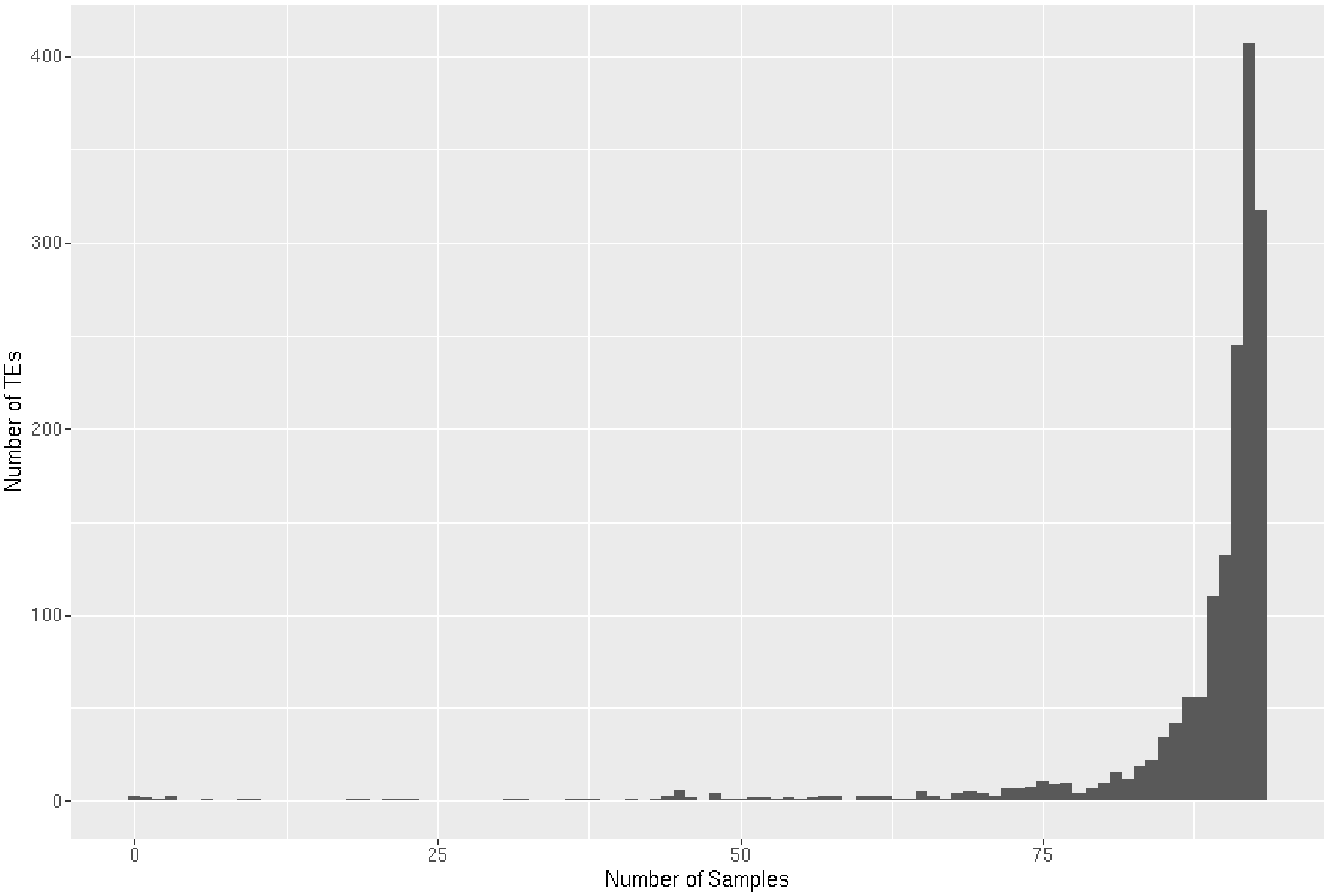

S10 Fig. Histogram showing the number of TEs (y axis) and the number of samples for which we were able to estimate its frequency.
