# Supplementary material for: Stress response, behavior, and development are shaped by transposable element-induced mutations in Drosophila
Source: PLoS Genet. 2019 Feb 12;15(2):e1007900. doi: 10.1371/journal.pgen.1007900 (PMC6372155; doi:10.1371/journal.pgen.1007900)
Supplement: S11 Fig — A) Zambia (Lack et al., 2015), B) France (Pool et al., 2012), C) DGRP (Raleigh) (Huang et al. 2014; Mackay et al. 2012), D) Italy (Bari) and E) Sweden (Stockholm) (Mateo et al 2018). All Pearson correlation coefficients r = 0.99 and p-value < 2.2e-16. (PDF) [file pgen.1007900.s011.pdf]

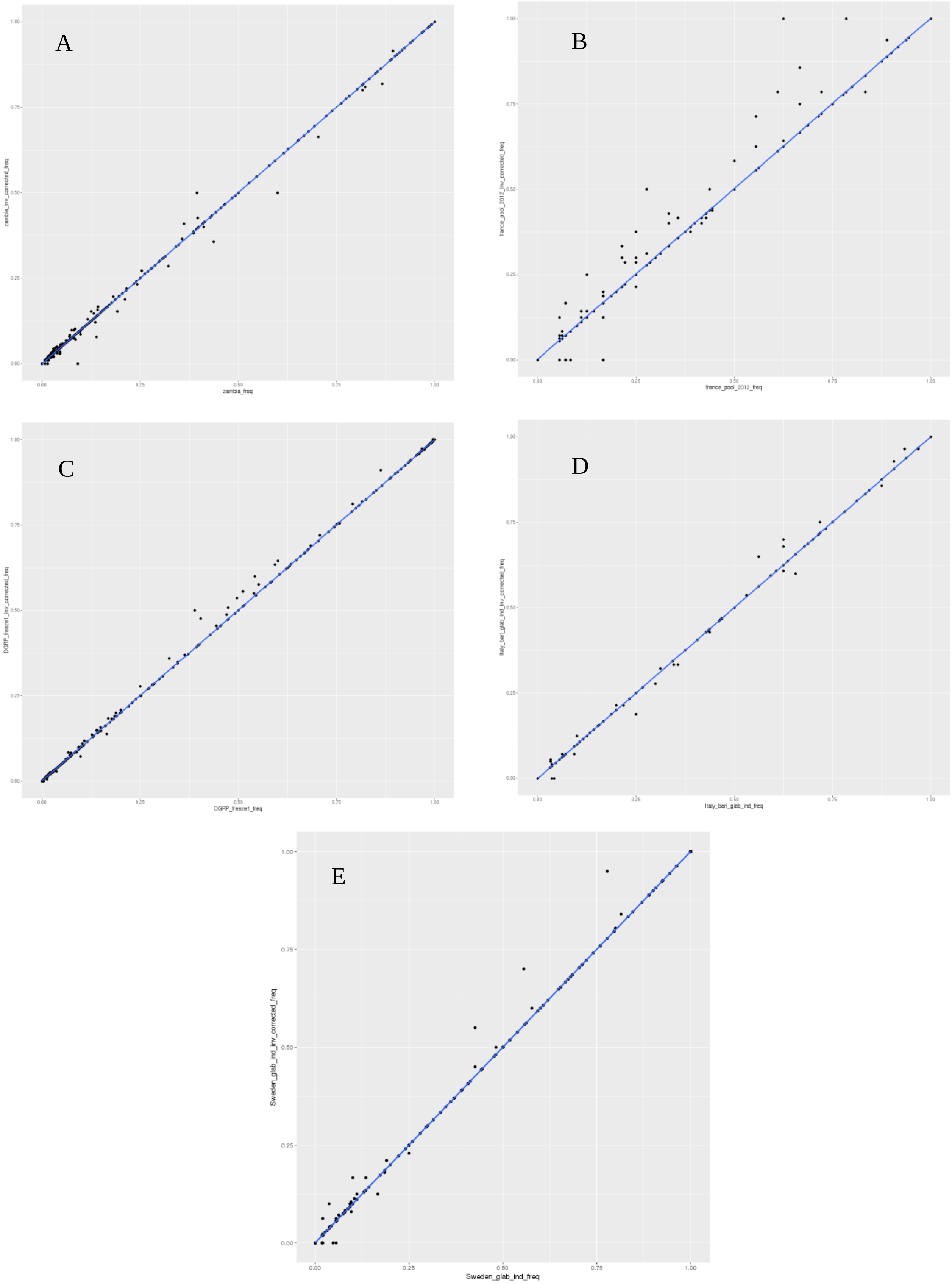

S11 Fig. TE frequencies estimated using all strains (x axis) vs. frequencies estimated after removing strains that contain inversions (y axis) for different individually-sequenced populations. A) Zambia (Lack et al., 2015), **B)** France (Pool et al., 2012), **C)** DGRP (Raleigh) (Huang *et al.* 2014; Mackay *et al.* 2012), **D)** Italy (Bari) and **E)** Sweden (Stockholm) (Mateo et al 2018). All Pearson correlation coefficients r=0.99 and p-value < $2.2e^{-16}$.
