# Supplementary material for: Stress response, behavior, and development are shaped by transposable element-induced mutations in Drosophila
Source: PLoS Genet. 2019 Feb 12;15(2):e1007900. doi: 10.1371/journal.pgen.1007900 (PMC6372155; doi:10.1371/journal.pgen.1007900)
Supplement: S12 Fig — A) Distribution of iHS values for all TEs and neutral SNPs. B) Distribution of iHS values for TEs and neutral SNPs at high frequency (> 0.10) in the OOA population (Raleigh) and in the African population (Zambia). C) Distribution of iHS values for TEs and neutral SNPs at high frequency (> 0.10) in the OOA population, but at low frequency in the African population. (PDF) [file pgen.1007900.s012.pdf]

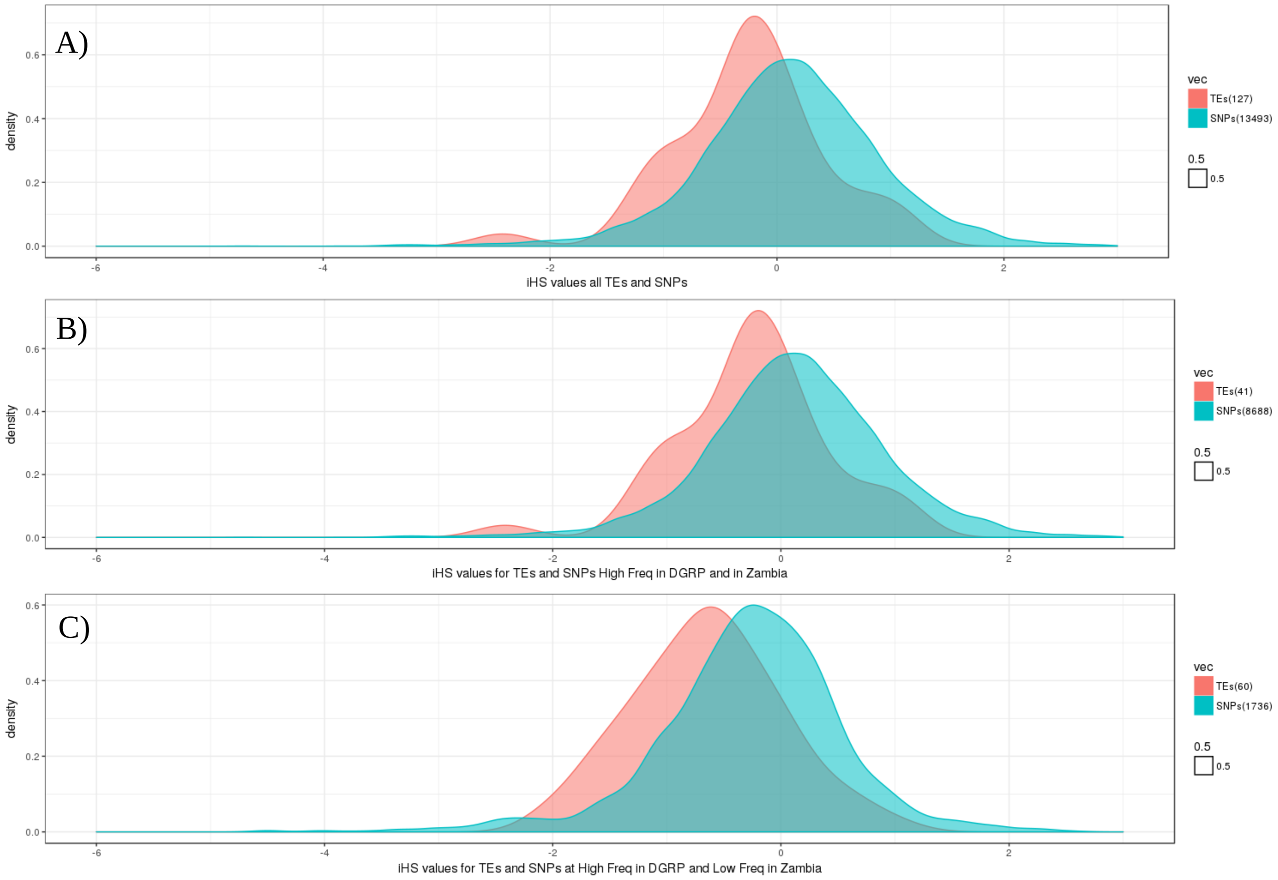

S12 Fig. Distribution of iHS values obtained for TEs (red) and neutral SNPs (cyan) in the North American population (DGRP, Raleigh, North Carolina). A) Distribution of iHS values for all TEs and neutral SNPs. **B)** Distribution of iHS values for TEs and neutral SNPs at high frequency (> 0.10) in the OOA population (Raleigh) and in the African population (Zambia). **C)** Distribution of iHS values for TEs and neutral SNPs at high frequency (> 0.10) in the OOA population, but at low frequency in the African population.
